# Specificity of oligonucleotide gene therapy (OGT) agents

**DOI:** 10.7150/thno.77830

**Published:** 2022-10-09

**Authors:** Daria D. Nedorezova, Mikhail V. Dubovichenko, Ekaterina P. Belyaeva, Ekaterina D. Grigorieva, Arina V. Peresadina, Dmitry M. Kolpashchikov

**Affiliations:** 1 Laboratory of Molecular Robotics and Biosensor Materials, International Institute SCAMT, ITMO University, 9 Lomonosov Str., St. Petersburg, 191002, Russian Federation.; 2 Chemistry Department, University of Central Florida, Orlando, FL 32816-2366, USA.; 3 Burnett School of Biomedical Sciences, University of Central Florida, Orlando, FL 32816, USA.

**Keywords:** gene therapy, therapeutic oligonucleotides, hybridization selectivity, cancer, off-target effect, antisense oligonucleotides, siRNA, miRNA, ribozymes, deoxyribozymes, CRISPR/Cas

## Abstract

Oligonucleotide gene therapy (OGT) agents (e. g. antisense, deoxyribozymes, siRNA and CRISPR/Cas) are promising therapeutic tools. Despite extensive efforts, only few OGT drugs have been approved for clinical use. Besides the problem of efficient delivery to targeted cells, hybridization specificity is a potential limitation of OGT agents. To ensure tight binding, a typical OGT agent hybridizes to the stretch of 15-25 nucleotides of a unique targeted sequence. However, hybrids of such lengths tolerate one or more mismatches under physiological conditions, the problem known as the affinity/specificity dilemma. Here, we assess the scale of this problem by analyzing OGT hybridization-dependent off-target effects (HD OTE) *in vitro*, in animal models and clinical studies. All OGT agents except deoxyribozymes exhibit HD OTE *in vitro*, with most thorough evidence of poor specificity reported for siRNA and CRISPR/Cas9. Notably, siRNA suppress non-targeted genes due to (1) the partial complementarity to mRNA 3'-untranslated regions (3'-UTR), and (2) the antisense activity of the sense strand. CRISPR/Cas9 system can cause hundreds of non-intended dsDNA breaks due to low specificity of the guide RNA, which can limit therapeutic applications of CRISPR/Cas9 by ex-vivo formats. Contribution of this effects to the observed *in vivo* toxicity of OGT agents is unclear and requires further investigation. Locked or peptide nucleic acids improve OGT nuclease resistance but not specificity. Approaches that use RNA marker dependent (conditional) activation of OGT agents may improve specificity but require additional validation in cell culture and *in vivo*.

## 1. Introduction

### 1.1. Oligonucleotide-based gene therapy (OGT)

Oligonucleotide-based gene therapy (OGT) is a variation of gene therapy that uses short synthetic DNA, RNA or their chemical analogs to hybridize to specific RNA or DNA targets followed by their inactivation. It is believed that OGT has a potential of combining the low immunogenicity of small molecule drugs with specificity and efficiency of target recognition by protein drugs (e.g. antibodies) 1. OGT has been under development for over 40 years 2. It aims at suppressing genes either responsible for the development of human diseases or interfering with conventional treatment (e.g. drug resistance). The OGT agents, subjects of this review, are antisense oligonucleotides (ASO agents), small interfering RNA (siRNA), ribozymes (Rz), deoxyribozymes (Dz) and CRISPR/Cas. To date, the global pharmaceutical market offers ten ASO agents and four siRNAs for the treatment of genetic disorders and the cytomegalovirus infection 3-6. However, despite significant progress in pre-clinical and clinical studies, not a single anti-cancer OGT agent has been approved for clinical use 5-7. The major problems in OGT development include inefficient intracellular delivery, lower efficiency, and high cost 2,3. This review analyzes yet another important issue, the specificity of OGT agents. Lack of drug specificity is a major cause of side effects associated with morbidity and mortality and increase health costs 8. In this review, 'specificity' is defined as the ability of an OGT agent to bind only a targeted RNA sequence in a complex mixture of biological molecules, such as those found in human body, without interacting with other biomolecules including non-targeted RNAs.

### 1.2. Overview: Hybridization dependent (HD) and hybridization independent (HI) off-target effects (OTE)

**Scheme 1:**
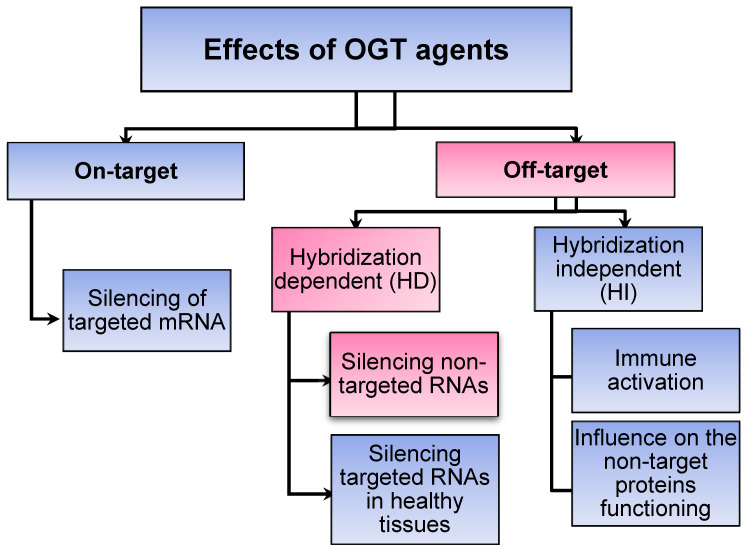
Classification of specific (on-target) vs non-specific (off-target) effects mediated by oligonucleotide-based gene therapy (OGT) agents 9. Sections highlighted in pink fall within the scope of this review.

OGT's off-target effects (OTE) can be classified in two broad categories: hybridization-dependent (HD) and hybridization-independent (HI) (Scheme 1) 9. HI OTE are referred to interactions of OGT with biomolecules (mostly proteins), which resemble the non-specific binding of small molecules to proteins. They are not associated with Watson-Crick base pairing. For example, phosphorothioate oligonucleotides (PS) are known to interact with a broad range of proteins causing cytotoxicity 10 or the immune system activation commonly observed for all OGT agents 11,12. ASO agents and Dz were found to activate proinflammatory response due to both non-natural chemical modifications and the presence of unmethylated CpG sequences. The later are recognized by immune system as components of bacterial pathogens via toll-like receptor-9 (TLR-9) 13. RNA-based OGTs (siRNA, Rz and CRISPR/Cas) can be recognized by immune system as viral RNAs followed by induction of the interferon-signaling pathways 14,15. Tracking HI OTEs is an important task since the therapeutic effect can be caused by the nonspecific action rather than by the targeted gene knockdown 16. On the one hand, non-specific immune response can be reduced by chemical modifications 17. On the other hand, immune activation can be beneficial for the treatment of cancers and viral infections 18.

HD OTEs are caused by suppression of unintended RNA targets with sequences possessing sequence homology to the targeted RNA (Scheme 1). A growing body of evidence for HD OTE have been accumulated in the last years. For example, only since 2014, but not earlier, HD OTE have been reported for ASO agents. Why there were no earlier reports on non-specific ASO agents? An overwhelming amount of evidence for low specificity of siRNA and CRISPR/cas is available. Can this problem create an obstacle for moving these technologies to therapy? To the best of our knowledge, there was no comprehensive review devoted to the analysis of fundamental sources and practical risks associated with the HD OTE. This work focuses on the analysis of HD OTE for the OGT agents to assess the scope of the problem and overview the available recipes for its solution. The review summarizes experimental data by agent type and analysis it with respect to the affinity-specificity dilemma.

### 1.3. The affinity-specificity dilemma

The hybridization specificity is a fundamental problem, known as affinity/specificity dilemma 19. Typical OGT binding site covers 15-25 nucleotides (nt) of targeted RNA. These lengths provide affinity sufficient to unwind secondary RNA structures and form a stable complex under physiological conditions. However, high affinity is achieved at the expenses of specificity 19. Indeed, under intracellular conditions, the stretch of 10 or more complementary nt is sufficient to form a stable complex. This opens an opportunity for OGT agents to bind multiple partially complementary non-targeted sequences. This low OGT specificity can cause HD OTEs *in vivo*. Designing OGT with low affinity to unintended RNA molecules remains a desirable but challenging task 9, 20-26.

Earlier, we proposed a general approach to solve the affinity/specificity dilemma, which takes advantage of multiple interactions between a target and a hybridization probe 27. This development has evolved into more complex nucleic acid-based sensors that can accomplish several target recognition tasks including the 'conditional activation' of OGT functions 23,27. Here we define 'conditional activation' as a generation of the OGT function under certain intracellular conditions, e. g. the presence of a cancer marker or viral RNA. This approach enables to render OGT inactive until encountering the specific RNA marker sequence, which activates the OGT function. This approach can reduce the HD OTE since the activity of OGT is controlled twice: at the stage of marker RNA binding and at the stage of targeted recognition. Moreover, the ability to target other than marker RNA sequence opens an opportunity to suppress genes vital for cell survival e. g. housekeeping genes 23. Therefore, separation of marker recognition and RNA knockdown functions may increase not only selectivity, but also the efficiency of OGT agents. In this review, we present examples of multicomponent and conditional OGT and discuss if the approach can add to the solution of HD OTE problem.

## 2. Antisense oligonucleotides (ASO agents)

**Figure 1:**
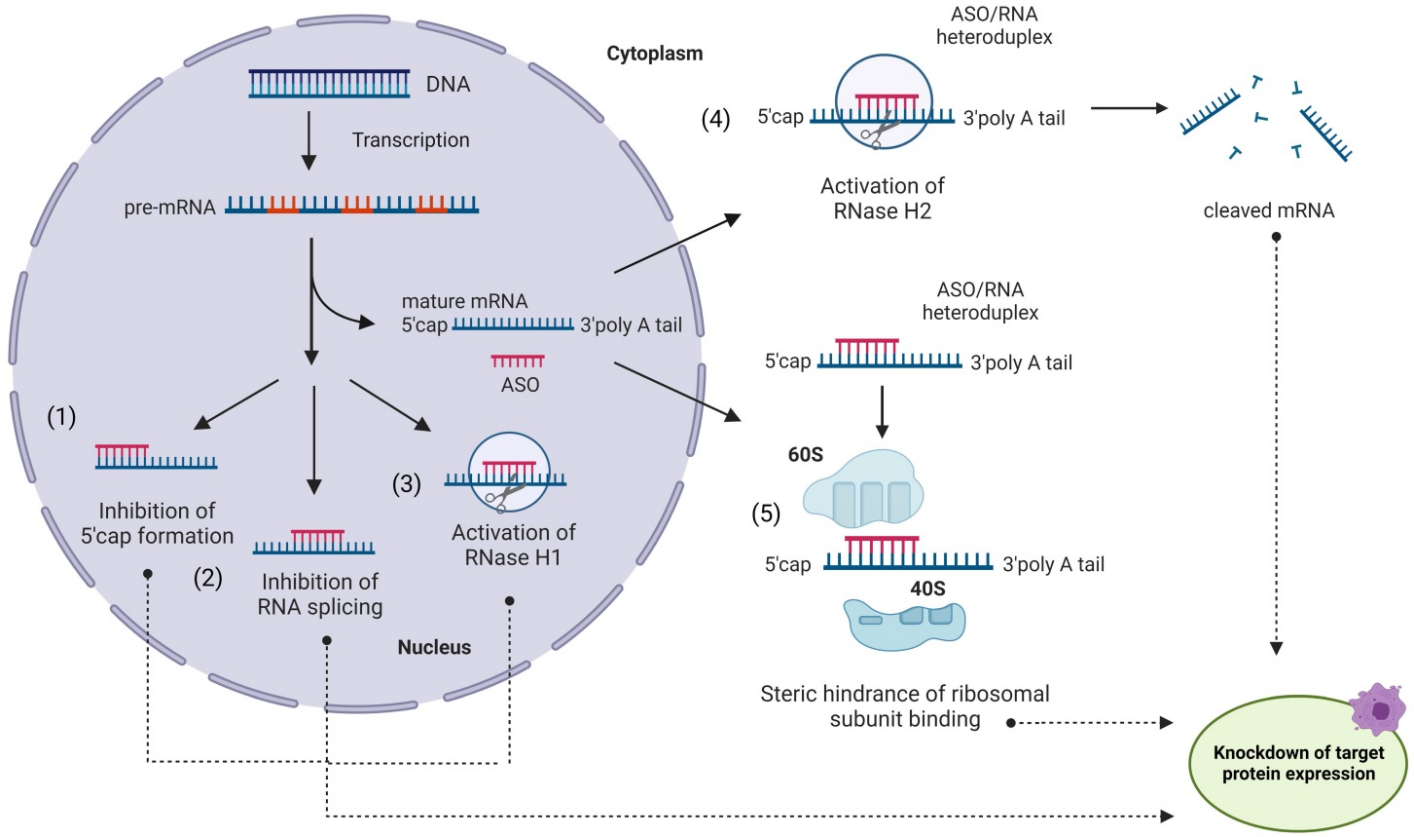
Antisense oligonucleotides (ASO) in action. An ASO internalized via endocytosis can bind a complementary mRNA fragment in the cytoplasm. Formation of the ASO/RNA heteroduplex induces activation of RNase H2 in the cytoplasm (4) and/or RNase H1 in the nucleus (3), leading to mRNA degradation 33. Alternatively, ASO can block the translation process without RNA degradation (5) by steric interference of ribosomal assembly. ASO can enter the nucleus and regulate mRNA maturation by preventing 5'-mRNA cap formation (1), inhibiting mRNA splicing (2) or (3) recruiting RNase H1 to pre-RNA cleavage.

Despite earlier related developments 28, 29, Zamecnik and Stephenson are commonly credited for introducing ASO principles in 1978 30. ASO agents are ~15-30 nt long synthetic single-stranded oligonucleotides complementary to mRNA targets 5, 31, 32 (Figure 1). Inhibition of translation can be achieved by one of the following strategies or their combinations: (i) RNase H-dependent mRNA degradation 33; (ii) splicing inhibition; (iii) translation modulation (Figure 1). Since RNase H hydrolyzes only RNA strands of ASO/RNA hybrids, multiple mRNA targets can be inactivated by a single ASO molecule. Theoretically, the ASO approach can selectively suppress any targeted gene 5,30-32. Importantly, ASO agents can target non‐coding RNAs 34. Inspired by this idea, tremendous efforts have been contributed to the development of ASO therapy during the last 44 years 5,31,32.

### 2.1. ASO chemistry and gapmers

**Figure 2:**
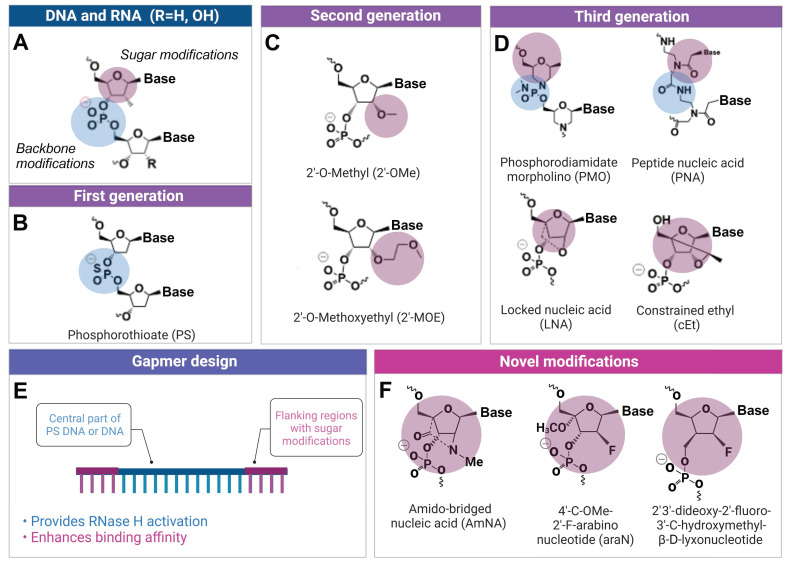
** Chemical modifications used in ASO and other OGT agents. A)** Natural deoxynucleotides and ribonucleotides. **B)** First generation phosphorothioate (PS) modified nucleotide (sulfur-substituted for a non-bridging oxygen of the phosphate group). **C)** Second generation 2'-O-Methyl (2'-OMe) and 2'-O-Methoxyethyl (2'-MOE) modified nucleotides (2'-hydroxyl group of RNA substituted with 2'-OMe or 2'-MOE). **D)** Third generation phosphorodiamidate morpholino (PMO), peptide nucleic acid (PNA), locked nucleic acid (LNA) and constrained ethyl (cEt) with various sugar and phosphate modifications. **E)** Gapmer antisense oligonucleotides, consisting of a DNA-based internal 'gap' and RNA-modified flanking regions (the most common are 2ʹ-OMe and LNA). **F)** Novel amido-bridged nucleic acid (AmNA), 4'-C-OMe-2'-F-arabinonucleotide (araN) and 2'3'-dideoxy-2′-fluoro-3′-C-hydroxymethyl-β-D-lyxonucleotide modifications (see 2.2.2 for details).

The development of ASO technology was accompanied by the evolution of chemically altered nucleotides resistant to degradation by natural nucleases. Other modifications were introduced to provide high affinity or improved specificity. First generation of ASO used phosphorothioate (PS) modifications, which enhanced nuclease resistance while maintaining RNase H-activation capabilities (Figure 2). PS ASO, however, displayed reduced affinity and hybridization kinetics compared to DNA, as well as exhibited elevated tendency of nonspecific binding to certain proteins that may also cause cytotoxicity 10. Second ASO generation with 2'-O-methyl (OMe) and 2'-O-methoxyethyl (MOE) groups (Figure 2) reduced toxicity and improved hybridization kinetics compared to PS DNA. Third ASO generation includes locked nucleic acids (LNAs), peptide nucleic acids (PNAs), constrained ethyl substituted (cEt) and phosphorodiamidate morpholinos (PMOs) modifications (Figure 2), which enhanced target affinity, nuclease resistance, biostability and pharmacokinetics.

Second and third ASO generations cannot stimulate RNase H activity. Therefore, hybrid oligonucleotide constructs named 'gapmers' were proposed to balance nuclease resistance and RNase H activation properties of ASO agents (Figure 2). Structurally, the gapmer design contains a central part of DNA or PS DNA (10-15 nt) flanked by 2'-OMe, 2'-MOE, LNA or cEt modified ribonucleotides (3-5 nt from both ends). In gapmers, the central part is sufficient to activate RNase H, while terminal modifications increase affinity to RNA targets. PMO and PNA are most frequently used for (ii) splicing inhibition or (iii) translation modulation (Figure 1), because they enable the highest target affinity among the available modifications. In addition, PMOs demonstrate reduced interactions with cellular proteins, metabolic stability, and absence of OTE 35. The affinity of the modified nucleotides to RNA increases in the following order PS < DNA < 2'OMe < MO < LNA 36. Based on this order, LNA should have lowest specificity, while PS - the highest according to the affinity/specificity dilemma 19.

New modified nucleotides are being introduced. For example, a non-gapmer ASO agents consisting of amido-bridged nucleic acid (AmNA, Figure 2F) were found to demonstrate a lower risk of hepatotoxicity 37. To enable RNAse H dependent cleavage, fluorine and 4'methoxy nucleotides were proposed (araN, Figure 2F) 38,39. Uracil and cytosine derivatives of 2'3'-dideoxy-2'-fluoro-3'-C-hydroxymethyl-β-D-lyxonucleotides (Figure 2F) incorporations are responsible for obtaining ASO agents molecules with reduced toxicities and OTE 39.

### 2.2. ASO drugs

So far, only a few ASO therapeutics have been approved for clinical use. The ten FDA-approved ASO agents include *Fomiversen* (brand name Vitravene^®^) FDA 1998, *Mipomersen* (Kynamro^®^) FDA 2013,* Eteplirsen* (Exondys 51^®^) FDA 2016*, Nusinersen* (Spinraza^®^) FDA 2016,* Inotersen* (Tegsedi^®^) FDA 2018, *Milasen* FDA 2018, *Golodirsen* (Vyondys 53^®^) FDA 2019, *Volanesorsen* (Waylivra^®^) FDA 2020, *Viltolarsen* (Viltepso^®^) FDA 2020, *Casimersen* (Amondys 45^®^) FDA 2021 4,5. More widespread usage of ASO is hindered, in part, by HI OTE. The Oligonucleotide Safety WorkIng Group (OSWG) recommends both computational and experimental assessment of HD OTE for ASO agents during drug discovery 9.

### 2.3.* In vitro* and* in vivo* hybridization dependent OTE

HD OTE have not been found for ASO agents until recently most likely due to the lower target affinity of the first-generation PS ASO 40. One of the first HD OTE was reported by Kakiuchi-Kiyota *et al.* for LNA gapmers in 2014 41. Microarray data revealed non-targeted suppression of the gene consistent with hepatotoxicity as well as 17 genes involved in the clathrin-mediated endocytosis 41. Kamola *et al.* found that PS-LNA gapmers designed against BACH1 transcription regulator also silenced multiple non-targeted RNAs in both exonic and intronic regions 42. Suppression single mismatched targets, in some cases, exceeded that of the intended target by several folds 42. Even two mismatches and a gap caused a significant knockdown of the four non-targeted genes 42. Authors attributed the observed HD OTE to the high ASO affinity to intronic sequences. This effect was not seen as a potential source of HD OTE prior this study. The correlation of ASO melting temperatures with the knockdown efficiency was found, which agreed with the affinity/specificity dilemma 19. It was concluded that given the observed tolerance for mismatches and the combined size of exons and introns, it is very difficult to design a potent OTE-free ASO ≤16 nt using currently available chemistries.

Furthermore, three independent studies reported hepatotoxicity of LNA and cEt gapmeric ASO agents in mice. The effect was attributed to the RNase H1-dependent knockdown of non-targeted pre-mRNA transcripts 43-45. RNA even with 3 nt mismatches could be suppressed by LNA gapmers due to their high binding affinity 43. Kasuya *et al*. showed that HD OTE could be accompanied by hybridization-independent innate immune response activation 44. Interestingly, no hepatotoxicity was found when ASO was replaced with siRNA targeting the same fragment of mRNA. This study provided the evidence of nucleus RNase H1 rather than cytoplasmic RNase H2 dependent suppression of non-targeted transcripts. Further, Hagedron et al. used two different gapmer ASO agents for non-overlapping regions of ApoB and Pcsk9 genes in mice to separate HD OTE from the events linked with downregulation of the target sequences 45. They concluded that off-target toxicity indeed was caused by binding of the gapmers to unintended RNA transcripts followed by RNase H1 degradation 45. A strong correlation of HD OTE and the ASO binding efficiency was found by Watt *et al*., who used 6 antisense oligonucleotides (ASO agents) and 832 nearly matched unintended transcripts 46. Likewise, Dieckmann *et al*. found correlation of 236 LNA-ASO's hepatotoxic potential with their HD OTE effect in different cell cultures. They demonstrated that LNA-ASO agents with T_m_ below 55 °C produced less HD OTE 47.

Recent study by Gentsch *et al.* showed that splicing and translation-blocking PMO ASO agents can cause HD OTE due to the high affinity to non-targeted sequences 20. Authors noted that PMOs hybridized to multiple RNAs with only 8-nt complementarity, which nevertheless blocked splicing and translation in *Xenopus tropicalis*. Moreover, non-Watson-Crick base pairs between guanine and thymine stabilized the PMO-RNA duplexes 20. In this case, optimization of the PMO concentration and binding affinity to unintended transcripts reduced but did not eliminate HD OTE 20. These studies provide evidence that the affinity/specificity dilemma persists *in vivo*: the higher the ASO affinity (presence of LNA or cEt modifications), the lower the RNA binding specificity and the higher the toxicity.

To date, there are no reports of HD OTE-dependent toxicity of ASO agents observed in clinical trials. Most likely, toxic ASO agents are eliminated during preclinical studies using animal xenograft models 43. Interestingly, despite hybridization dependent toxicity shown in *in vitro* and *in vivo,* several gapmer ASO agents were approved by FDA and currently are in clinical trials 4,5. This is probably because side-effects are dose-dependent, and systemic treatment with ASO is generally well tolerated. Dose-limiting toxicities include thrombocytopenia, hypotension, fever, and asthenia 32. The links between these symptoms and HD OTE are likely but have not been experimentally established yet.

### 2.4. Strategies to reduce hybridization dependent OTE

The explored strategies include (1) selection of ASO variants by sequence alignment algorithms to assess binding against non-targeted RNA; (2) control of ASO binding affinity by optimization the length and chemical modification of ASO. We also discuss below the possibility of using highly selective multicomponent ASO. It is worth reiterating that an important stage in developing therapeutic ASO agents is preclinical studies, which experimentally eliminate toxic ASO candidates without studying the mechanism of their toxicity.

#### 2.4.1.* In silico* and *in vitro* ASO analysis

The most common strategy for reducing ASO HD OTE is the assessment of the number of partially complementary sites in a replisome of a given cell type/organism and defining regions complementary to their target RNA 48. Lindow *et al.* proposed a step-by-step strategy for selection of therapeutic candidates: (i) sequence database interrogation, (ii) microarray analysis to identify potential off-target transcripts *in vitro,* and (iii) detailed study of preclinical toxicity *in vivo* 9. Yoshida *et al.* used *in silico* analysis to find binding sites in human mRNA for several thousands of hypothetical ASO agents that form one or several mismatches. The number of partially complementary regions was found to increase with the growing number of the tolerated mismatches 49. However, the presence of non-targeted complementary regions does not necessarily cause OTE *in vivo* as ASO binding efficiency depends on accessibility of the partially complementary RNA fragments, as well as on the number and types of mismatches. The position of mismatches may also affect the OTE, although the correlation between the mismatch positions and knockdown efficiencies is unclear 49. Holgersen *et al*. experimentally evaluated the performance of *in silico* screens for off-target splicing events of 81 ASO and found a false discovery rate of astonishing 99%. The authors concluded that currently used in silico methods have limitations for predicting HD OTE and experimental screening is preferred 50. Scharner *et al.,* found multiple mis-splicing events for one of the ASO agents tested and reached the same conclusion: 'off-target effects are difficult to predict' 51.

#### 2.4.2. Controlling ASO binding affinity by changing length and chemical modifications

It was advertised that LNA-modified ASO agents with higher than DNA affinity to targeted RNA have 'remarkable specificity' 52, 53. This statement contradicts the affinity/specificity dilemma (higher hybridization specificity is only possible for the cost of lower affinity) 19. The high specificity claim is also confusing in the view of numerous reports of HD OTE found predominantly for LNA-containing ASO agents (section 2.3. of this review). Below we explain this contradiction.

You *et al.* refer to the ability of short LNA ASO to differentiate single nucleotide variations (SNV) in targets superior to all-DNA ASO 52. However, the optimization of ASO agents for SNV differentiation was not done in this study. Moreover, the criteria of selectivity used: the temperature range in which the SNV is differentiated, was not relevant to practical ASO use. More investigations are needed to establish the relative ability of all-DNA and LNA-modified ASO to differentiate SNV, especially in RNA targets folded in secondary structures. Theoretically, it is possible that optimal LNA ASO have better SNV differentiating activity than optimal all-DNA or all-PS ASO. This is because optimal LNA ASO should be shorter than optimal all-DNA or all-PS ASO, while in short hybrids a single mismatch should add a greater destabilization than in longer ones 52. However, to best of our knowledge this has not been demonstrated so far. The SNV differentiating ability, however, is different than 'specificity' typically measured for ASO as an ability to differentiate a single targeted RNA from the transcriptome (see section 2.3). Indeed, it was experimentally shown that using 16-nt long LNA gapmers with reduced binding affinity (T_m_ < 55 °C) can mitigate HD OTE regularly observed for longer ASO gapmers 50,51. The downside of this strategy is that the 16-nt ASO may not provide sufficiently long stretch of nucleotides to bind a unique sequence in a complex RNA mixture of a transcriptome size. Indeed, Yasuhara decreased HD OTE by extension ASO gapmer from 14 to 18 nt 54. This data demonstrates that optimization of ASO size and the number of LNA nt provides a tool for finding balanced ASO sequence with moderate affinity and moderate specificity but does not resolve the affinity/specificity dilemma 19. ASO modified by other artificial nucleotides 55 or conjugated with ASO/RNA complex stabilizing groups 56,57 should experience the same fundamental challenge.

Importantly, in contrast to LNA ASO, there was no HD OTE reported for low-affinity PS ASO. Reducing affinity of LNA ASO by shortening or mixing with PS nucleotides or UNA nucleotides could be used to find the affinity/selectivity balance in ASO agents 58.

#### 2.4.3. Binary ASO

**Figure 3:**
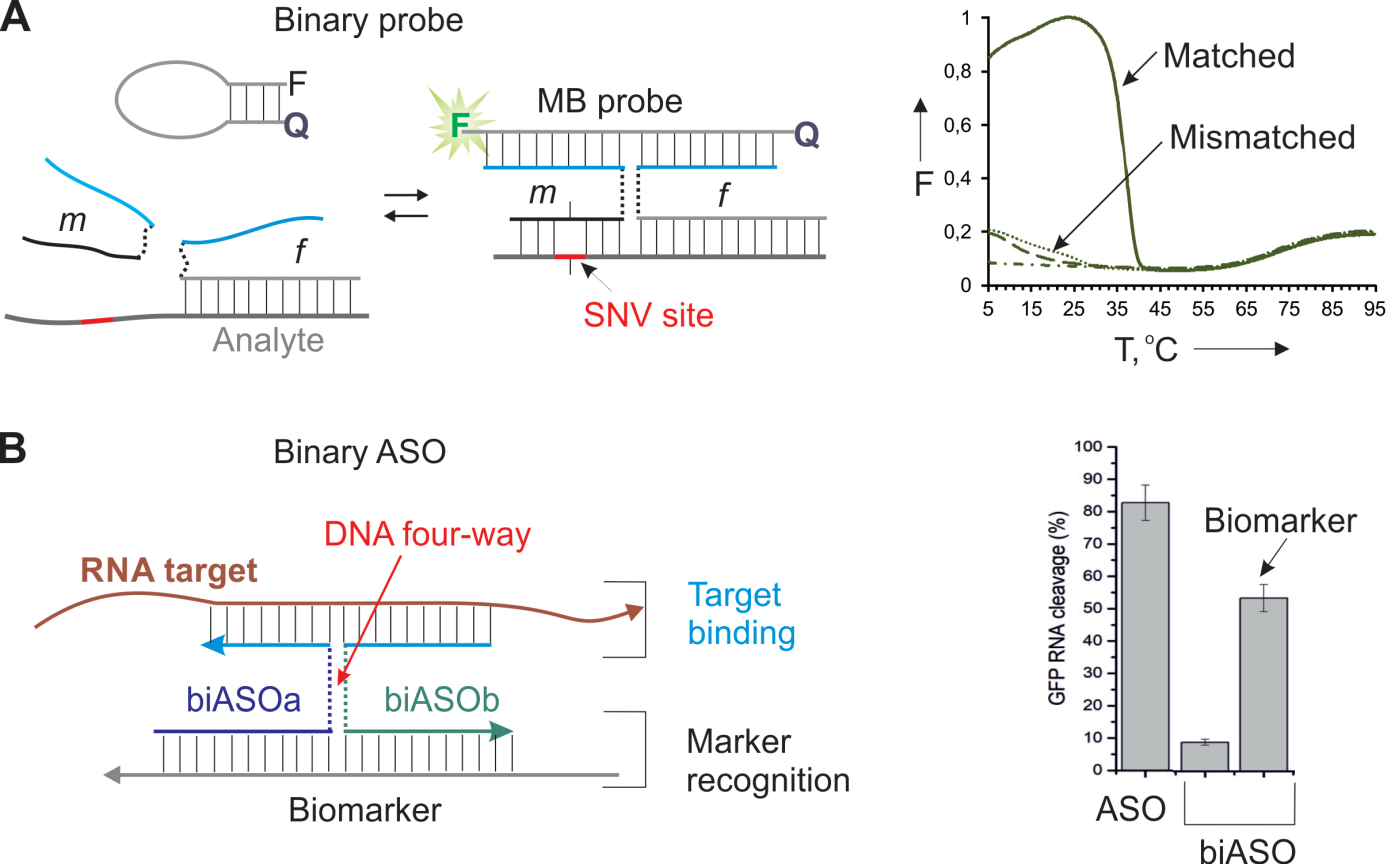
** X probe for highly specific recognition of nucleic acids. A)** Strands m and f hybridize to both the MB probe and the targeted analyte to form a fluorescent complex stabilized by a DNA four-way junction structure 60. Right panel: melting profiles of the fluorescent complexes in the presence of fully matched and mismatch analyte. Dash-dotted line - the MB probe alone; Dashed line - the binary probe without analyte present 61. **B)** Binary ASO consists of biASOa and biASOb strands that form a 4WJ complex with a targeted RNA only in the presence of a biomarker strand. Right panel: RNase H-dependent cleavage of the targeted RNA by biASO in comparison with the traditional ASO 26.

A well-acknowledged approach to achieve high sequence specificity under physiological conditions is using binary hybridization probes 59. In this approach a target is recognized by two probes cooperatively before the recognition event takes place. One example of such approach is DNA four-way junction or X probe (Figure 3) 60. It takes advantage of two DNA strands (m and f Figure 3A) and a molecular beacon (MB) probe. Both f and m strands contain fragments complementary to the MB probe and the target. Strand m has a short (7-12 nt) analyte-binding arm that forms a stable complex only with the fully matched target under ambient or near physiological conditions. Importantly, the overall length of the target recognition region is > 20 nt, which ensures binding of a unique site in the entire transcriptome. In the presence of a fully matched target, strands m, f and the MB probe form a 4-stranded complex, in which the MB probe acquires an elongated highly fluorescent conformation. It was demonstrated that the probe has unprecedented ability to differentiate single-base variations in the range of 5-41 °C (Figure 3A, right) 61.

Recently, we adopted the X probe approach for the conditional activation of ASO agents (Figure 3B) 26. In this approach, strands biASOa and biASOb recognized a cancer biomarker sequence with high specificity followed by binding targeted mRNA and its RNase H-dependent degradation. The marker recognition and target binding functions are separated in this design, which makes it possible to recognize the biomarker sequence (cancer marker miRNA or viral RNA), while suppressing another RNA (e. g. a housekeeping gene mRNA for efficient cell death). The biASO approach demonstrated ~ 30% lower RNA degradation activity in comparison with the traditional monolith ASO, but excellent specificity toward the biomarker sequence 26. The reduced efficiency can potentially be compensated by targeting an appropriate (most vulnerable) gene. The high specificity of the approach in cell culture remains to be validated.

## 3. RNA interference; siRNA and shRNA agents

Small interfering RNAs (siRNA) are 21-23 nt dsRNA with 2-nt overhangs on the 3' ends of both strands. They can be used as exogenous OGT agents taking advantage of the natural RNA interference (RNAi) mechanism. The RNAi pathway first described for *C.elegans* 62 was later discovered in plants and mammals 63. Since then, RNAi has become a widely used tool for gene knockdown in biomedical research due to its greater efficiency and predictability than the ASO approach 64.

**Figure 4:**
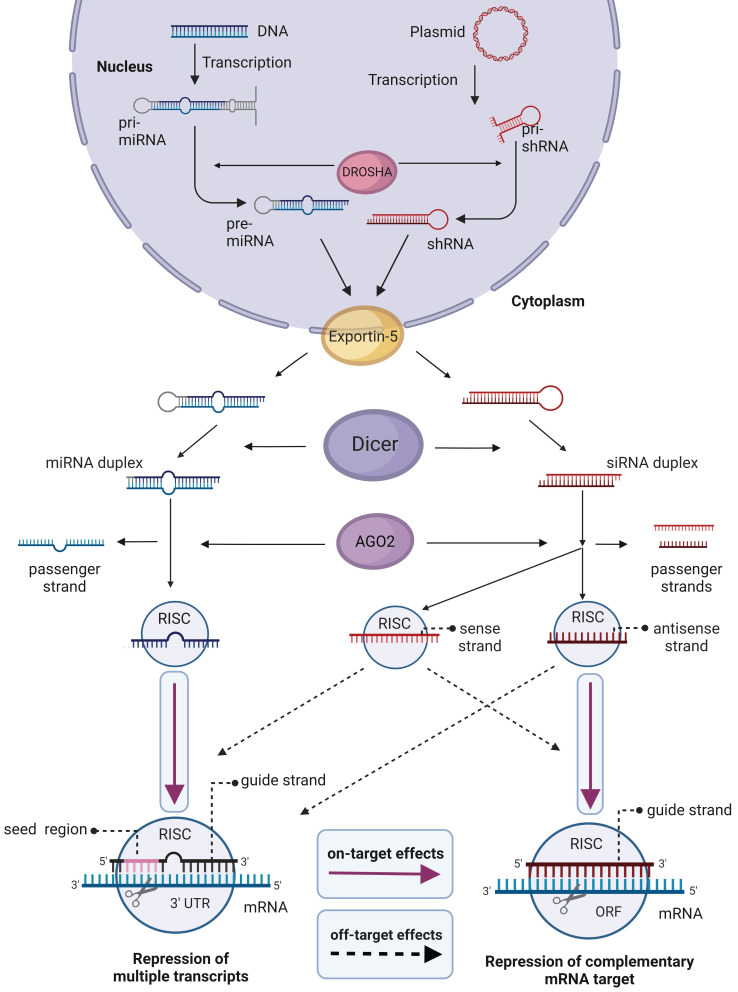
Biogenesis and effects of siRNA and miRNA in cells. In the nucleus, Drosha endoribonuclease cleaves primary miRNA (pri-miRNA) and the precursor of siRNA (pri-shRNA) to yield 70-100 nt pre-miRNA or shRNA, which are then transported to the cytoplasm by Exportin 5. In the cytoplasm, the RNAs are converted to 18-25 nt bp miRNA or 21-23 bp siRNA by Dicer. For gene silencing and therapy, chemically synthesized mature 19-24 bp siRNA or miRNA can be delivered into cells. Alternatively, cells can be transformed with plasmids coding for pri-shRNA or pri-miRNA that maturate using the same mechanism. RNAi silencing process starts in the cytoplasm by association of siRNA or miRNA with the RNA-induced silencing complex (RISC). Argonaute 2 (AGO2) component of RISC complex unwinds dsRNA and nicks the passenger sense strand. However, this action is not selective, and the antisense strand of siRNA also may be removed, thus using the sense strand as a guide. The guide strand of siRNA in the active RISC complex binds mRNA target causing its cleavage. RISC/miRNA complex binds 3'-untranslated regions (3' UTR) of mRNA with perfect complementarity in the seed region (2-8 nt site) causing down-regulation of multiple mRNAs. Active RISC/siRNA complex can silence non-targeted mRNA by miRNA-like mechanism. Magenta arrows show specific effects, dashed arrows show HD OTEs.

Natural mechanisms for siRNA maturation and posttranscriptional gene downregulation are shown in Figure 4 65. Generally, short hairpin RNA or small hairpin RNA (shRNA) serve as precursors of siRNA. Once exported into the cytoplasm, shRNA is cleaved by Dicer endoribonuclease to produce mature siRNA. Next, the RNAi process starts with the association of siRNA with the RNA-induced silencing complex (RISC). After the guide (antisense) strand is activated, RISC complex recognizes and binds an mRNA target followed by its cleavage and degradation of the mRNA fragments by cellular exonucleases. The activated RISC complex stays effective for multiple rounds of mRNA degradation 65, which is the foundation for high efficiency of the RNAi mechanism.

Micro RNA (miRNA) uses a similar maturation and gene knockdown mechanisms (Figure 4). However, miRNAs usually target 3'-untranslated regions (3'-UTR) of multiple transcripts (Figure 4). It seems to be impossible to design a miRNA against one specific gene due to homology of 3'-UTR regions of many mRNA 66. Therefore, nowadays miRNA is not widely used as a therapeutic agent 5, 67. In turn, siRNA and shRNA target mRNA coding sequences and thus can be tailored to knockdown unique genes. Therefore, they are used for therapeutic purposes, for the identification of new members of cellular pathways 68, and in the search of new therapeutic targets 69.

The two main sources of siRNA- and miRNA-mediated HI OTE are (1) inducing the interferon-activated pathways and expression of antiviral cytokines through the Toll-like receptor 3 (TLR3) and TLR7/8 14; (2) saturating the miRNA machinery, which inhibits processing of endogenous miRNA. The later affects Exportin-5 70-72 and the RISC complex (Figure 4) 73,74. While the 1^st^ problem is common for all OGTs, the 2^nd^ is siRNA/shRNA specific. Despite challenges associated with OTEs, there are four siRNAs approved drugs agains genetic diseases: *Patisiran* (FDA 2018, amyloidosis); *Givosiran*, (FDA 2019, porphyria); *Lumasiran* (FDA 2020, primary hyperoxaluria type 1); *Inclisiran* (FDA 2021, hypercholesterolemia) 3. In all clinical trials, siRNA/shRNA demonstrated common adverse effects including fever, fatigue, and nausea. Dose-limiting toxicities for each type of therapeutic agent are reviewed in 75, 76. The association of these adverse effects with specific RNAi mechanisms has not been established yet.

### 3.1. siRNA/shRNA mediated hybridization dependent OTE

There are two main sources of HD OTE for siRNA and shRNAs: (1) the sense strand of the siRNA mistakenly used as a guide by RISC that may cause suppression of non-targeted genes; (2) miRNA-like targeting the 3'-UTR of non-targeted mRNAs 77-90.

In the first investigation of the siRNA off-target activity, insulin-like growth factor 1 receptor (IGF1R) and mitogen-activated protein kinase 14 (MAPK14) were used as targets 80. It was found that sixteen anti-IGF1R and eight anti-MAPK14 siRNAs suppressed several non-targeted transcripts having various length of complementarity regions to both sense and antisense strands of siRNA (Table 2). Moreover, siRNA against KPNB3 and FLJ2029 also silenced non-intended MAPK14 due to 11 and 14 nt complementarity, respectively 80. HD OTE found in this study was related to both sense and antisense strand-mediated silencing with complementarity to the unintended transcripts of at least 9 nt 80.

miRNA-like HD OTE is the main cause of non-desired gene suppression. It is caused by binding 3'-UTR by the seed region (positions 2-8 from the 5' end) of the guide strand 81-85. In a large-scale knockdown experiment, Lin *et al.* found that two siRNAs designed to suppress heterodimeric transcription factor (HIF-1) downregulated either GRK4 or BTK due to 7 nt sequence identity in the seed region with 3' UTR of hif-1α mRNA 81. Further, Jackson *et al.* designed six siRNAs against MAPK14, MPHOSPH1, PIK3CB, SOS1 genes and the shRNA against PLK1 82 and found downregulation of multiple non-targeted transcripts due to partial complementarity to their 3' UTR (Table 2) 82. Even though changes in the seed region reduced the set of original off-target transcripts, they caused down-regulation of yet other transcripts, thus making it impossible to reduce OTE simply by adding mismatches in the seed region 82. Birmengham *et al*. confirmed the key role of the miRNA-like mechanism of HD OTE targeting three genes - PPIB, MAP2K1 and GAPDH (Table 2) 83. At the same time, they found that the number of Watson-Crick base-pairs between non-target mRNA to siRNA contributes moderately to HD OTE, except almost perfect matches 83. Further, Nielsen *et al.* proved that expression of non-targeted mRNA decreased log-additively with the increase of the seed match length 84. Investigation of siRNAs against Apolipoprotein B in mice confirmed emergence of miRNA-like OTEs *in vivo* (Table 2) 85. These OTEs overlapped with those obtained *in vitro* in murine hepatoma cells but differed from those demonstrated in human liver-tumor derived cells 85. These results suggested that such effects are species-specific despite overlapping seed matches of human and mouse cells 85.

Importantly, analysis of siRNA-mediated HD OTEs may contribute to interesting findings. For example, Lin* et al.* discovered that the Mcl-1 gene plays a key role in cancer cell resistance to a small molecule inhibitor ABT-737 86. From 4000 siRNAs, they found three 'top hit' siRNAs, causing apoptosis of the inhibitor-resistant cells treated with ABT-737. All three hits silenced the Mcl-1 gene by miRNA-like mechanism (Table 2) thus revealing the importance of this gene for overcoming the ABT-737 resistance 86. In another study, Schultz *et al.* analyzed the OTE of a 6,000-siRNA library. They found 172 siRNAs downregulating at least one of the two transforming growth factors (TGF)-β receptors 1 and 2 (TGFBR1 or TGFBR2) by the miRNA-like OTE 87. Moreover, the TGFBR2 mRNA had multiple silencing sites at the 3'UTR, suggesting that this gene could be regulated by endogenous miRNAs 87. Based on these findings, three miRNAs (miR-20a, miR-34a and miR373) were identified as endogenous inhibitors of TGF-β receptor 2 (TGFBR2) 87. In a study of the role of nucleostemin in human glioblastoma cancer stem cells (GBM-CSCs), one shRNA against nucleostemin caused HD OTE leading to apoptosis of both GBM-CSCs and non-stem glioma cells 88. Remarkably, use of this shRNA reduced tumorigenic potential of GBM-CSCs in nude rats, showing its potential for fighting CSCs 88. Although this study could not identify primary shRNA targets, it was found that the treatment mostly affected MAPK kinase pathways and suggested that the primary target might be a transcription factor involved in one of these pathways (Table 2) 88. Further, by analyzing false-positive effects caused by miRNA-like OTEs, Adams *et al.* discovered a new transcription factor MYBL1 that regulated E-cadherin (CDH1) expression known to participate in epithelial mesenchymal transition 89. Researchers developed an approach called si-Fi, to identify how a particular OTE of an siRNA library affect each given gene expression 90. SENSORS allows classification of off-target transcripts into positive and negative by their effect on gene expression. MYBL1 was ranked as a highly negative off-target transcript and proved to be a transcriptional factor for CDH1 90.

While analyzing HD OTE, Putzbach *et al.* found that a set of siRNA and shRNA against the death receptor CD95 and its ligand CD95L can cause cancer cell death by downregulating several survival genes (Table 2) 91. Researchers proposed to separate this feature of RNAi into a specific group named 'death induced by survival gene elimination' (DISE) and use it as a novel approach for cancer therapy 92. Furthermore, it was shown that shRNAs and siRNAs targeting CD95L caused cancer cell death with high selectivity in murine ovarian cancer model without affecting healthy cells 93. This is most likely connected with the occupancy of healthy cell RNAi machinery by endogenous miRNA, that prevents activation of the DISE 92,93. Today, three DISE patents are awaiting approval by the US Patent and Trademark Office 94-96.

In conclusion, numerous well documented evidence of HD OTE for siRNA have been accumulated so far. First evidence appeared soon after the introduction of the technology indicating the ubiquity of siRNA HD OTE. The HD OTE are dominated by the miRNA-like mechanism i.e. partial complementarity of the miRNA seed region to the 3'UTR of non-targeted mRNA by both antisense and sense strands of siRNA.

### 3.2 Attempts to improve selectivity of siRNA and shRNA

#### 3.2.1. Chemical modifications

Several reports suggested chemical modifications to improve siRNA specificity. For example, 2'-OMe modifications at the 2^nd^ position of the siRNA guide strand reduced both the number of off-target transcripts with the 3'UTR matches and the magnitude of their downregulation 97. The mechanism of this effect was associated with conformational changes in the RISC complex caused by the 2'-OMe modification, which led to a weaker binding of the imperfectly matched transcripts 97. Addition of a single modification (e. g. phosphorylation, 5'-O-methylation 98,99, unlocked nucleoside analogs (UNA) 100, 5′-*O*-methyl-2′-deoxythymidine and 5′-amino-2′, 5′-dideoxythymidine 101 at the 5'-end of the sense strand prevents its loading into RISC followed by the increase of the antisense strand activity. A single UNA 100 in the seed region and single nucleotide bulge at position 2 (from the 5' end) of the antisense strand 102 were found to reduce miRNA-like off-target silencing with no loss in efficiency. This was attributed to the improved RISC's differentiation of the targeted from non-targeted transcripts 102. In addition, recent studies of Kobayashi *et al*. demonstrated *in vitro* and *in silico* that 2'-OMe modification of nucleotides 2-5 of the sense strand contributes to avoiding miRNA-like HD OTE while maintaining the on-target activity 103, 104.

#### 3.2.2. Careful design and experimental controls

To further improve the specificity of siRNA, the following should be considered at the stage of siRNA design: (i) the content of immunostimulatory GU motifs; (ii) possible binding to the non-targeted transcriptome through BLAST, and (iii) matches with the 3'-UTR of non-target transcripts. To assist in the last task, Birmingham *et al.* 83 developed a web-based search tool to track 3'UTR hexamer seed matches for any given siRNA. Dongen *et al.* suggested Sylamer, an algorithm for detecting miRNA target and siRNA off-target signals in 3′-UTR from a ranked gene list 105,106.

Petri and Meister detailed how to avoid siRNA OTE by experimental design 107. They suggested using siRNA at the lowest possible concentrations, as well as using siRNA controls targeting mRNA that are not expressed in the chosen biological system or having 'random' sequences. In addition, using a pool of independent siRNA sequences targeting different regions of the same mRNA ensures specificity of the observed inhibitory effect. Another way to verify siRNA specificity is to perform rescue experiments by expressing a recombinant equivalent of the targeted gene from a vector using the open reading frame unaffected by the siRNA under investigation 107.

#### 3.2.3. Altering siRNA structure

Structure of siRNA impacts its ability to cause OTE. Various siRNA architectures were suggested to avoid loading of the passenger (sense) strand into RISC. For example, it was shown that asymmetric absence of only one overhang nucleotide at the passenger strand promotes preferential loading of the antisense strand into RISC and reduces the passenger strand induced OTE 108. Another strategy uses small internally segmented siRNAs (sisiRNAs), which are three-stranded associations of one antisense and the sense strands split into two 10-12 nt fragments 109. Further, it was found that simple shortening of the sense strand with preserved overhangs improves the antisense strand specificity and reduces the OTE 110, 111. Short sense strand was not loaded in RISC, which reduces both saturation of the RNAi machinery and the passenger strand-mediated HD OTE 109-111.

It was found that a synthetic 25-30 bp RNA duplex named 'Dicer substrate interfering RNA (DsiRNA)' can be up to 100 times more efficient than traditionally designed siRNA 112. The enhanced potency of the longer duplexes was explained by the interconnection of the Dicer-dependent DsiRNA processing with the subsequent siRNA incorporation in the RNAi complex. DsiRNA favors selection of the antisense strand as a guide for RNAi machinery 113. DsiRNA against the Myc gene has passed Phase 1 clinical trials for cancer treatment 114. However, DsiRNAs have also been found to induce miRNA-like HD OTE 91.

An alternative strategy is the siRNA 'dual-targeting design', which does not eliminate but rather takes advantage of the passenger strand to be used as a guide by RISC. The dual-targeting design uses both strands to silence two different targets and, consequently, eliminates the OTE caused by the passenger strand 115.

Another way to avoid loading of the sense strand into RISC is to use the Dicer-independent RNAi mechanism 116. This technology was inspired by miR-451 that uses Ago2 enzyme for maturation and does not require Dicer for the processing. Several structures of such agents were reviewed by Herrera-Carillo and Berkhout 116. In brief, there are several miR-451-mimicking RNAi agents that can be expressed from a plasmid or be directly delivered into the cytoplasm. All of them are RNA hairpin structures with 16-19 bp stems and 2-5 nt loops 116. They use Ago-2 enzyme and poly(A)-specific ribonuclease (PARN) for cleavage and trimming the 3'-end to yield the mature product that is ready to be loaded into the RISC. This technology has been used as an antiviral treatment. Short shRNA (sshRNA) against hepatitis C virus (HCV) showed inhibition of viral replication without significant hepatotoxicity in mice 117. The Ago2-dependent shRNA (AgoshRNA) against human immunodeficiency virus 1 (HIV-1) were used with the 3' terminal hepatitis delta virus (HDV) ribozyme and demonstrated high potential of AgoshRNA technology *in vitro* and suggested to be used instead of shRNA 118. Recently, a detailed approach for designing the 3' HDV ribozyme-fused agoshRNA was published 119. In addition, the plasmid expressing agoshRNA together with Ago-2 enzyme were used to silence genes in malaria parasite *Plasmodium berghei,* the organisms that lack canonical RNAi machinery 120.

#### 3.2.4. Conditional siRNA release

Activation of RNAi by RNA triggers opens an opportunity to directly silence genes in cells containing specific RNA markers, (e.g. cancer markers). This strategy can reduce the unwanted side effects since the RNAi silencing will be triggered only in the presence of a marker RNA.

**Figure 5:**
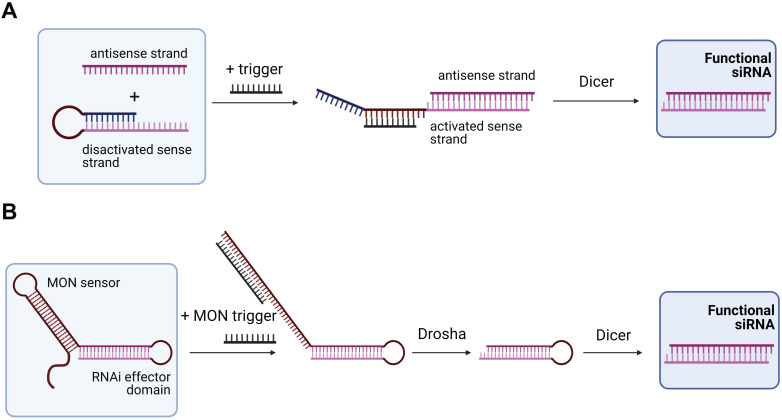
** Trigger-dependent siRNA formation. (A)** Activable siRNA probe. The first proposed technology for activation of the siRNA therapeutic function by an RNA trigger. In the initial stage, the sense strand is disactivated by forming a hairpin structure that can be opened by the trigger strand with subsequent binding to the antisense strand. The activated complex is than processed by Dicer with the yield of an active functional siRNA molecule 121. **(B)** Modified oligonucleotide-inducible RNAi (MONi RNAi). Double hairpin structure expressed from a plasmid contains two stem-loops: (1) MON sensor that recognizes a small chemically modified oligonucleotide (MON) trigger, and (2) RNAi effector domain. After recognizing the MON trigger, MON sensor opens and makes the RNAi effector domain available for further processing by Drosha and Dicer with the yield of a functional siRNA molecule 122.

In 2009, Masu *et al.* suggested using an RNA trigger for siRNA production 121. They used a sense strand closed into the hairpin structure with the loop complementary to the RNA trigger (Figure 5A). Binding of the trigger RNA to the hairpin released the sense strand for the binding with the antisense strand followed by processing by Dicer yielding an active siRNA 121. It was found that a 19-nt regulatory stem domain (blue in Figure 5A) prevents siRNA formation in the absence of the trigger strand. Addition of the trigger reduced activity of the targeted firefly luciferase from 91 to 38%. Next, Kumar *et al.* improved this design by using a plasmid expressing a modified oligonucleotide-inducible RNAi (MONi-RNAi), which folds in a double hairpin structure consisting of the sensor stem-loop (MON sensor) and the RNAi effector domain (Figure 5B) 122. MONi-RNAi expressed in the nucleus binds a small oligonucleotide trigger (MON trigger), thus producing a hairpin RNA available for Drosha processing 122. Bujold *et al.* designed a DNA-cube with encapsulated siRNA that could be released in the presence of an RNA trigger 123.

**Figure 6:**
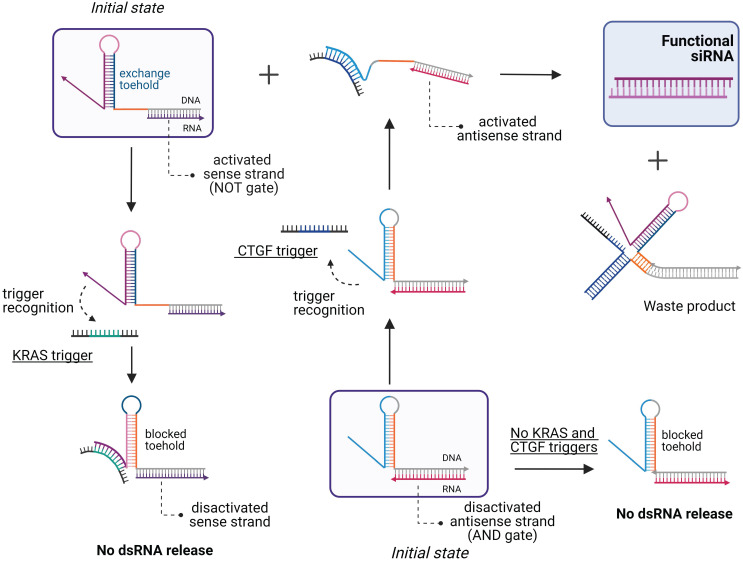
** Scheme of the logic gates for the multi-trigger RNA/DNA hybrid system.** The system is comprised of a 3-input AND gate and a NOT gate that are constructed by coupling the sense hybrid (activated by the connective tissue growth factor (CTGF) that used as an RNA trigger) with the antisense hybrid (designed to repress the strand exchange in the presence of a trigger sequence derived from the Kirsten rat sarcoma proto-oncogene (KRAS) mRNA). Both hybrids and the CTGF trigger are required for the dsRNA release, while the presence of the KRAS trigger inhibits strand exchange 22.

In 2013, Hochrein *et al.* engineered five small conditional RNAs (scRNA) to silence an RNA target (Y) only in the presence of an mRNA target (X). They used several RNA hairpins as in the work of Masu *et al.* 121 and a simple logic for the formation of DsiRNA or shRNA in response to the detection of the mRNA target followed by processing the complex by intracellular enzymes yielding active siRNA 124. In 2019, Zakrevski *et al.* developed four logic gates for trigger-inducible or repressible siRNA release (Figure 6) 22. The undoubted efficiency of this strategy in extracellular experiments was not supported by cell experiments. Afonin *et al.* designed “multi-trigger” hybrid system based on RNA-DNA hybrids releasing active dsRNA upon meeting each other in cellular cytoplasm 125. The activity of this system was demonstrated both in cells and *in vivo* 125. In 2021, Gong *et al.* developed a smart multiantenna for miRNA triggered siRNA activation using a hybridization chain reaction amplification machine 25. Researchers placed such RNAi prodrug in extracellular vesicles and demonstrated therapeutic effect both *in vitro* and *in vivo* 25. Thus, there is a high probability of success of the RNA inducible siRNA release in further *in vivo* studies. However, the potential of such systems to reduce HD OTE have not been studied yet.

In conclusion, the siRNA approach suffers from both HI and HD OTEs. The HD mechanisms include activation of RISC by both sense and antisense strands and by the miRNA-like OTE. Although researchers are trying to improve the RNAi specificity, using chemical modifications, accurate design and altering the siRNA structure, none of these approaches can solve the full set of problems. The development of RNAi agents that uses both Dicer-dependent and Dicer-independent mechanisms seems to be the most powerful strategy to avoid major HD OTEs. Activation of RNAi by intracellular RNA molecules may further increase the specificity due to triggering of the knockdown only in the presence of specific markers but requires additional studies. Combination of the approaches promises to mitigate the OTE of siRNA agents.

## 4. Ribozymes and Deoxyribozymes

**Figure 7:**
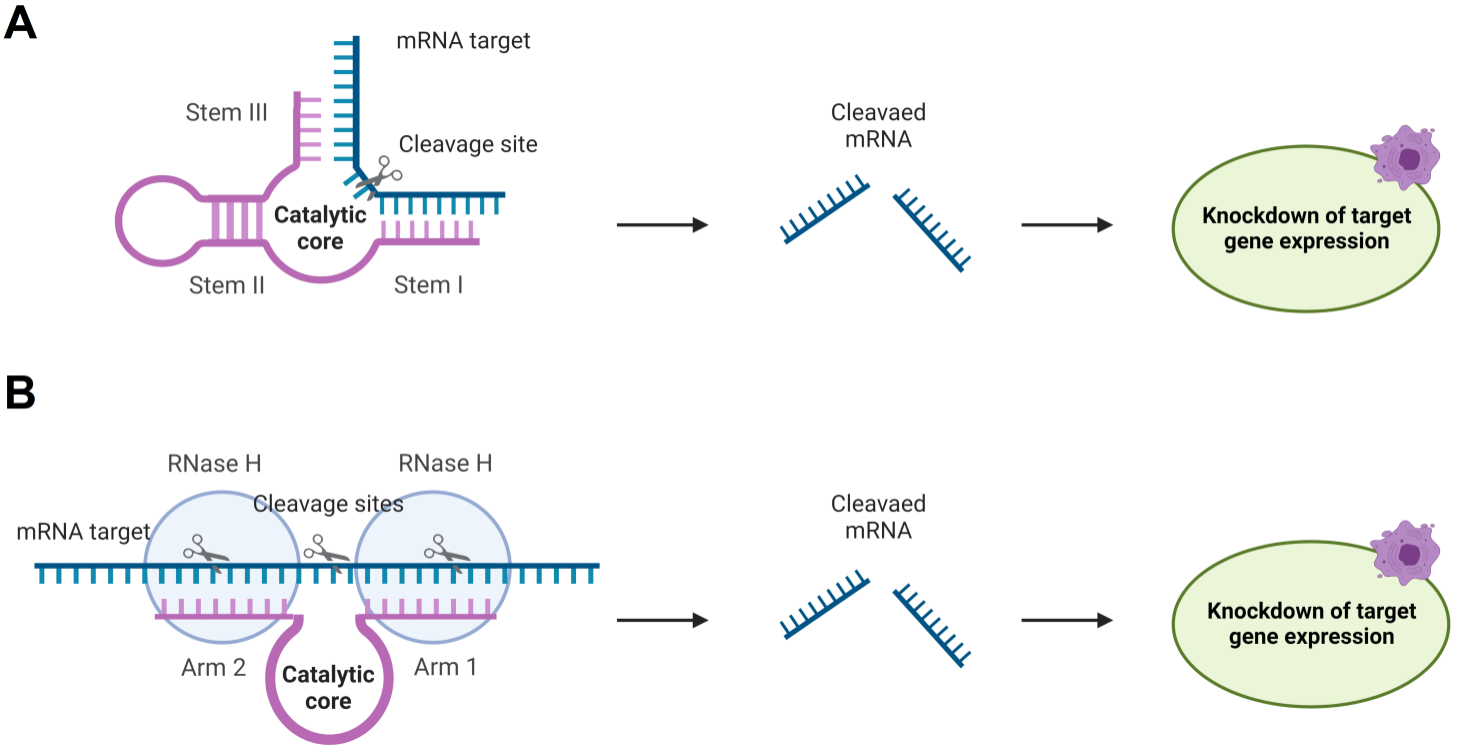
** Rz and Dz for gene knockout. A)** Hammerhead Ribozyme (Rz) (magenta) binds mRNA (blue) by two RNA-binding stems I and III followed by its catalytic cleavage, which leads to inhibition of the target expression. **B)** Deoxyribozyme (Dz) 10-23 hybridizes to mRNA targets by two RNA binding arms 1 and 2 forming two stretches of RNA-DNA hybrids. The RNA can be cleaved by the Dz itself or by RNase H.

RNA-cleaving catalytic RNA (ribozymes, Rz) and DNA (deoxyribozyme, Dz) possess nucleotide sequences (catalytic core) responsible for the lyase activity and RNA-binding fragments (stems I and III, and arms 1 and 2 in Figure 7). Rz and Dz are attractive agents for gene silencing due to their ability to cleave RNA in protein-independent manner. This unique future leaves room for a broad spectrum of chemical modifications of Dz and Rz preserving their catalytic cores. Moreover, allows incorporating Dz and Rz in complex functional associations (see section 4.3.2 for examples). Rz and Dz agents have been reported to be more specific in binding RNA targets 126. Protein independence may contribute to the high specificity. Indeed, RNA-cleaving machinery (e. g. RISC complex or RNases H) can stabilize the complexes of OGT agents with their RNA targets 124. A stable complex reduces the specificity in agreement with the affinity/specificity dilemma 21. On the other hand, Rz and Dz bind RNA targets by two relatively short RNA-binging arms (8-12 nt), which can be adjusted to form stable complex only with fully matched RNA target. This design resembles the principles of binary probes 59.

### 4.1. Ribozymes' hybridization dependent OTEs

Rz are natural catalytically active RNA structures discovered in 1982 127. Relatively simple and small Hammerhead (Figure 7A), Hairpin and hepatitis delta virus Rz have been considered as gene silencing agents 128, 129. These self-cleaving ribozymes were reengineered for intermolecular 'trans' cleavage 130.

Hammerhead Rz (HHRz) (Figure 7A) is the smallest and the best characterized catalytic RNA-cleaving Rz 131. Although HHRz appears to be less effective than siRNA, they offer advantages due to their specificity without any reported OTE in cells 132. However, detailed investigation of the HHRz specificity *in vitro* demonstrated its ability to cleave 3'-truncated targets with as little as 3-nt base-pairing 133. This lack of specificity can be explained by high affinity of Rz to RNA substrates 133. On the other hand, HHRz was found to provide high specificity for single-base mismatches and for truncation at the 5'-end 134. Another study demonstrated that HHRz targeting a fused TEL-AML1 chimeric RNA in the 8 nt fusion site can cleave not only the targeted RNA, but also unfused AML1 RNA, which had a 7-nt fragment complementary to the RNA-binding arm and a 5-nt mismatch at the 5'-end of RNA 134. However, high specificity was achieved with a redesigned stem III of HHRz by removing 4 nt complementarity to the AML1 RNA in the middle of the stem thus hybridizing to HHRz 134. These two studies indicate that HHRz can be highly specific only in a certain range of RNA-binding arm's lengths, and its design requires screening for possible non-specific targets. Several investigations including clinical trials did not observe any evidence of clinically significant adverse effects of HHRz 135-138.

### 4.2 Deoxyribozymes' hybridization dependent OTEs

In 1994, Roland R. Breaker and Gerald F. Joyce isolated the first RNA-cleaving Dz 140, which was followed by the selection of Dz 10-23 and 8-17 in 1997 141. The later had catalytic rates of RNA cleavage higher than that of Rz reengineered for silencing purposes. Other advantages of Dz over Rz include lower synthetic cost, greater chemical and biochemical stabilities, as well as a greater spectrum of chemical modifications available from commercial vendors. Moreover, unlike Rz, Dz can trigger RNase H antisense-like RNA cleavage, which can result in improved efficacy inside cells. This opened the era of Dz application in biotechnology. Majority of the Dz gene knockout agents were designed based on Dz 10-23 and 8-17 due to their small size and high catalytic activity 7.

Several hybridization-independent HI OTEs (also shared by Dz with ASO agents) have been identified: (i) non-specific binding to proteins due to different chemical modifications, e.g phosphorothioates, 142; (ii) aptamer-like binding of proteins; (iii) activation of toll-like receptors (TLRs) and other components of the innate immune system 18. The issue of interaction with TLRs can be solved by using extra additional Dz agents as controls. These controls are designed to bear single-nucleotide mutations in the 15-nt 10-23 catalytic domain (i.e., 5'-GGC TAG^6^ CTA CAA CGA-3', G^6^>C^6^), which render the Dz inactive in cleaving but near identical in all other aspects. Another way to improve specificity is to test for TLR9/NF-kB activation alongside reference oligonucleotides 143. Different *in vitro* and *in vivo* studies provided contradictory data about OTEs of Dz targeting c-Jun HI 144, 145. However, no clear evidence of HD OTE for Dz has been published yet. Moreover, both clinical trials with Dz targeting c-Jun or EBV-LMP1 Dz have shown no OTE 146,147. A recent study has summarized data for the Dz clinical trials 7. Table 3 summarizes the available data on application of Dz in cancer treatment.

Rz and Dz are the least impacted by HD OTE among all OGT agents. Dz were shown to selectively cleave only the mutant allele leaving the wild type unaffected 150, 151. Other studies have demonstrated specific cleavage of a chimeric mRNA leaving the native mRNA intact 134, 152. The major obstacle towards Dz clinical applications is their low gene knockdown efficiency resulted from either low affinity to the target or insufficient cellular uptake 126, 153.

### 4.3. Attempts to improve specificity of ribozymes (Rz) and deoxyribozymes (Dz)

#### 4.3.1. Computational selection of Rz and Dz sequences

General rules for designing HHRzs GT agents were summarized by Sallivan 154. Computational analysis of the catalytic core parameters and their dependence on the targeted sequences, named Rz's fingerprints, were used to predict intracellular activity of HHRz 155. It was found that interaction between stems I and stem II increases activity of HHRz *in vitro.* This interaction should be preserved during *in silico* design of highly active HHRzs 155. In 2016, a computational tool for HHRzs design, named RiboSoft, was reported 156. This approach was found to be effective against a mutant version of the PABPN1 gene mRNA *in vitro* and *in vivo* 157.

When developing therapeutic Dz agents, researchers usually use the rules described in the pioneering work of Santoro and Joyce 142. More recent work by Ahmadi *et al*. contains a detailed explanation of the design of a Dz against a bacterial β-galactosidase gene using bioinformatics tools 158. Even though the results of the developed Dz activity were not presented, the described approach might become useful in designing therapeutic Dz agents. Most recently, Mohammadi-Arani *et al*. published a web application for the design of RNA- and DNA-cleaving Dz, named DNAzymeBuilder 159. The algorithm uses an internal database and provides as and outcome a list of Dz sequences to carry out the cleavage reaction, optimal reaction conditions, the expected yield, and the reaction products.

#### 4.3.2. Conditional Rz activation

**Figure 8:**
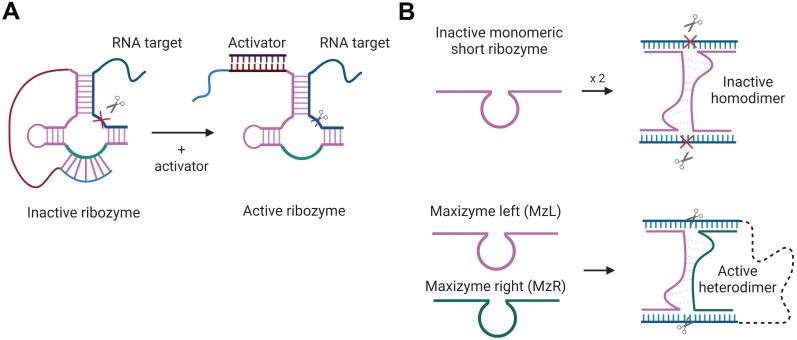
Rz-based constructs for target-dependent activation of RNA-cleaving functions. A) HHRz-based targeted Rz-attenuated probe (TRAP) 160. The light blue fragment interacts with the green fragment of the HHRz catalytic core in the “closed” inactive state. The activator sequence (dark purple) is complementary to the red Rz fragment so that their binding opens the cleavage core and activates the Rz. An RNA target is shown in navy blue; the cleavage site is indicated by the scissor's signs. B) Maxizyme technology 161: minimized HHRz and HHRz homodimer (top) have no cleavage function, while the heterodimer (bottom) consisting of two Rz strands (MzL and MzR), binds two RNA fragments and cleaves both sites immediately.

Activation of Rz- and Dz-cleaving function in the presence of specific nucleic acids sequences was proposed. The first strategy is based on HHRz, TRAP - targeted ribozyme-attenuated probe 160. TRAP is an Rz sequence that has a 3'-terminal «attenuator», which sequesters the catalytic core thus inactivating the Rz (Figure 8A). The sequence of the activator binding leads to the opening of the attenuator-cleavage complex, thereby activating the RNA cleavage function. Another technology, named Maxizyme, represents a heterodimer of inactive minimized HHRzs that can cleave two different target sites with high specificity (Figure 8B). Maxizyme can form an active conformation and cleave the target only when it binds two sites in the target mRNA. This supposed to increase specificity of mRNA recognition. Maxizyme was able to specifically cleave chimeric bcr-abl mRNA *in vitro* and in mice 161. Despite positive initial results, both TRAP and Maxizyme technologies were not widely accepted possibly due to low stability towards cleavage by nucleases.

#### 4.3.3. Conditional Dz activation

**Figure 9:**
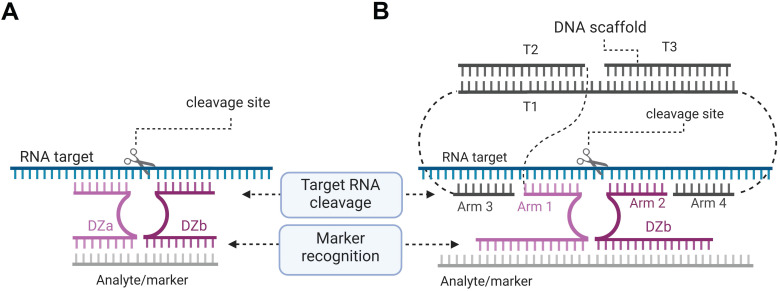
** Dz 10-23 constructs for the cancer marker-dependent activation of the RNA cleavage function. (A)** Binary Dz design for cancer treatment. Dza and Dzb strands bind a complementary nucleic acid analyte (grey line) and re-form a catalytic core of Dz, which cleaves an RNA target. If the Dzb strand has a single nucleotide mismatch with the analyte sequence, the construct fails to cleave the RNA target due to the instability of the Dz core in such a partial construct. **(B)** DNA machine for cancer therapy. Dza and Dzb strands hybridize to the cancer marker sequence (grey) and form a catalytic core that can bind and cleave a housekeeping gene mRNA (“RNA target”, cyan).

We proposed to use an RNA marker activated Dz for cleaving vital housekeeping genes exclusively in cancer cells 23. This was achieved by separating the cancer marker recognition function from the RNA-cleaving function (Figure 9) 162. In this approach, the parent Dz was split into Dza and Dzb strands, which formed a catalytic Dz core only when hybridized to the cancer marker RNA. The active core cleaved another targeted RNA (e.g. mRNA of a housekeeping gene). The approach demonstrated high selectivity of marker recognition with somewhat reduced cleavage efficiency in comparison with the non-split Dz 10-23 162.

Multicomponent probes enable both high specificity and tight binding of nucleic acid analytes despite their stable secondary structures 163, 164. By using these principles, we designed an association of three DNA stands (T1, T2 and T3) to collectively constitute a DNA nanomachine with the following functions: 1) recognition of a cancer marker sequence; 2) binding and unwinding folded targeted RNA using arms 3 and 4; 3) binding the cleavage site high selectivity by arms 1 and 2; and 4) RNA cleavage. The DNA machines use several short binding arms rather than one long (15-30 nt) monolith sequences, which is expected to have little or no nonspecific RNA binding when applied in cells. We demonstrated that the cancer maker can be cut out from the longer RNA marker sequence by using two additional Dz agents so that this shorter RNA product can be then used as an activator for cleavage of the targeted RNA 165. Recently, the binary Dz technology was integrated into a rigid DNA nanostructure named 'Nanotweezer', which was used for cleavage of specific mRNA in living cells 166, thus proving that this technology can be applied under intracellular conditions.

Dz agents are less promising GT agents than siRNA or ASO due to lower gene silencing efficiencies. The efficiency could be limited by low Dz affinity to folded RNA targets inside cells or by low Mg^2+^ concentration and/or by the instability of Rz and Dz core to nuclease degradation 126. Indeed, ASO and siRNA can be protected from nuclease degradation by either chemical modifications or by forming complexes with the protein machineries (e.g RNase H or RISC). In contrast, protein machinery independent Dz or Rz are not protected from nuclease degradation. On the other hand, chemical modifications of the catalytic core nucleotides may reduce Dz activity and thus have only limited application 167, 168.

## 5. CRISPR/Cas principles and the origin of side effects

**Figure 10:**
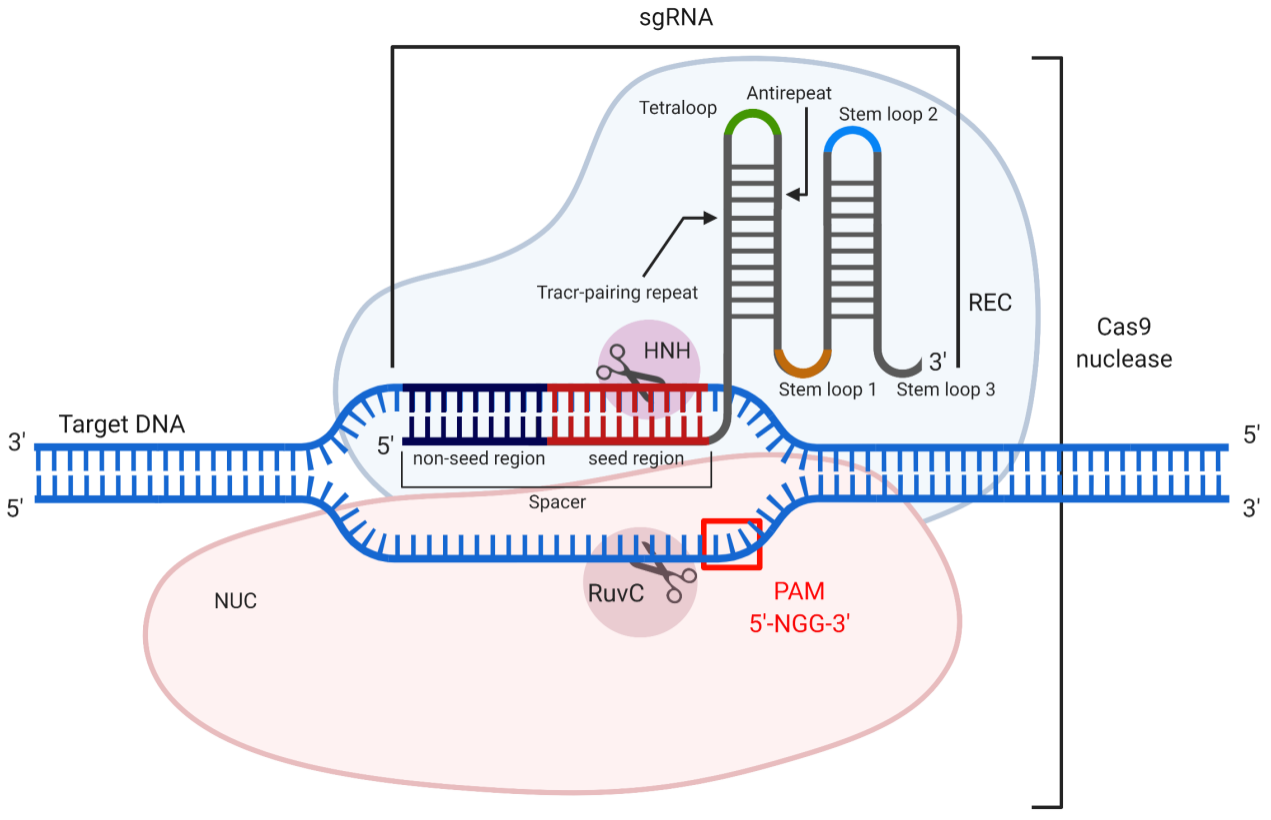
** CRISPR/Cas9 mechanism of action.** The guide RNA (sgRNA) in association with the recognition (REC) lobe specifically hybridizes with the target dsDNA. Hybridization occurs through the spacer sequence, containing seed and non-seed regions. Cleavage of the dsDNA occurs only if PAM (5'-NGG-3') is presented, which is recognized by the nuclease (NUC) lobe. After recognition of the target dsDNA sequence and its PAM, the domains HNH and RuvC introduce a double-stranded break (DSB).

CRISPR (clustered regularly interspaced short palindromic repeats) and CRISPR associated (Cas) nucleic acid technology (CRISPR/Cas) is considered an attractive tool for therapeutic gene 169. The most well-studied example is CRISPR/Cas9 found in *Streptococcus pyogenes* 170. CRISPR/Cas9 is used to cleave double-stranded DNA (dsDNA) to silence genes, to add a new gene fragment or change the original one. Cas9 is an endonuclease that uses a guide RNA (gRNA) or an artificially modified single guide RNA (sgRNA), whose “spacer” region is complementary to a specific dsDNA fragment (Figure 10). The 3'-terminal 10-12 nt fragment of the spacer (called the “seed region”) determines the specificity of Cas9/dsDNA interaction, where the presence of a single mismatch between the DNA and the spacer aborts the Cas9 action 171 (Figure 10). To confirm foreign origin of the dsDNA, the Cas9 nuclease recognizes a NGG sequence (where N is any nucleotide) called 'protospacer adjacent motif' (PAM), located in 3'-end after the spacer sequence. As a result, binding of the sgRNA/Cas9 complex to the dsDNA and recognition of PAM are followed by the hydrolysis of both DNA strands with HNH and RuvC domains of Cas9 causing a double-stranded break (DSB). In cells, the DSB is then repaired by either the non-homologous end joining (NHEJ) or homology directed repair (HDR) mechanisms. NHEJ provides knockout of the targeted gene, while HDR enables insertion of a new DNA strand into the space of the DSB 171. Technologically significant analogs of the described system include CRISPR/Cas12a, which recognizes a different PAM sequence and generates products with sticky ends after DNA cleavage 172, and CRISPR/Cas13, which cleaves RNA targets rather than DNA 173 thus making them analogous to the OGT agents discussed above.

HI OTEs of the CRISPR/Cas systems include non-specific PAM recognition and the effects caused by different CRISPR/Cas delivery methods 174-176. The PAM-related OTE are associated with the ability of Cas9 to recognize not only the NGG site but also the NAG sequence 171, 176. For minimizing the PAM-associated OTEs, Cas9 orthologues have been used. For example, Cas9 of *Streptococcus thermophilus* (StCas9) and *Staphylococcus aureus* (SaCas9) were required for a more complicated and specific PAM: NNRNVA and NNGRRT (where R = G or A, and V = G, C or A) 176. Another example is Cas12a and its orthologues, which bind to the TTTV PAM sequence 172. The CRISPR/Cas system is a relatively large RNA/protein association, which requires sophisticated intracellular delivery vehicles. The delivery methods can contribute to OTEs. For example, viral vectors (AAV) and lipid nanoparticles are popular as delivery vehicles for *in vivo* therapy but can cause humoral immune response and unspecific cytotoxicity 177, 178. The use of plasmids in cell therapy leads to a long-term production of Cas9 components and can also cause immune response 179. It was reported that such side effects can be avoided by using Cas9 in the form of RNPs (ribonucleoproteins) packaged in less toxic cationic lipid particles or viral particles 179.

### 5.1. CRISPR/Cas hybridization dependent OTE

HD OTEs of CRISPR/Cas9 system are mostly associated with the nonspecific binding of gRNA to the DNA target. They include an OTE-associated chromosomal damage.

#### 5.1.1. Off-target interaction between gRNA and DNA targets

The review by Zhang *et al*. is one of the earlier discussions that a large number (>50%) of HD OTEs are associated with the interactions between mismatch-containing gRNA and dsDNA 174. Fu *et al.* observed Cas9 activity even when gRNA had mismatches with the DNA in the non-seed regions 180. In addition to mismatch-tolerant gRNA/DNA interactions, Lin *et al.* noted the OTE due to the formation of nonspecific DNA/RNA bulges at the hybridization sites. When studying the cleaving activity of Cas9 in the HEK293T cell line, 114 potential off-target sites were identified, 15 of which had a 45.5% mutation probability 180. Recently, Wessels and group investigated the mismatch tolerance of Cas13 with GFP containing HEK293 cell lines. Single mismatches in the position of the 10th nucleotide of Cas13 gRNA spacer were tolerated for RNA-cleavage 181.

#### 5.1.2. Chromosomal mutations and lesions

The most detrimental OTEs that limit the therapeutic use of CRISPR/Cas are chromosomal rearrangements and lesions resulting from NHEJ. This type of damage appears even with completely on-target interactions of the system with the targeted dsDNA. Ghezraoui's group noted rare cases when Cas9-mediated chromosomal damage was up to several hundred nucleotides long. NHEJ repair was found to lead to the tumor-forming chromosomal translocations 182. Other studies reported large chromosomal deletions and rearrangements 183, 184. More recently, Zuccaro's group have carried out a large-scale study of the effect of mutations at the EYS gene locus in human embryos. The study revealed that the allele-specific chromosome loss occurred in both targeted and non-targeted DNA 185. These *in vitro* studies demonstrate that the use of CRISPR/Cas9 *in vivo* can cause significant non-targeted alterations in genome. These alterations are the consequences of low specificity of the gRNA/targeted DNA hybridization under intracellular conditions.

### 5.2. Attempts to reduce hybridization dependent OTE

The described off-target effects already can be addressed through computation-assisted design of the gRNA sequence and the use of engineered Cas9 nucleases or their natural orthologs.

#### 5.2.1. Bioinformatic-assisted gRNA design

Since the beginning of CRISPR/Cas era, many online services and software products have been developed for both the gRNA design, selection of DNA targets and predictions of on- and off-target interactions. The Root laboratory investigated interactions of 1841 sgRNAs with six mouse genes and three human genes 186. The support vector machine (SVM) online tool was developed for the design of highly active sgRNA sequences 186. The model includes analysis of each nucleotide in the 30-nt targeted DNA fragment and its GC content. In parallel, the Liu's laboratory has developed a method for predicting on-target effects using Elastic-Net 187. The Church laboratory developed an SVM-based on-target scoring model that compares two libraries of the targeted sites and sgRNA. The models described above have been used to predict on-target effects in such software and online tools as E-CRISP, CHOPCHOP, PROTOSPACER, CLD, CRISPOR, CRISPETa, GuideScan and Guide Picker 188. Simultaneously with the prediction of the on-target effects, algorithms for predicting OTE were also developed. Zhang and his team evaluated interactions of > 700 gRNA variants with > 100 off-target loci. The data revealed a correlation between the off-target interactions and the number, position, and distribution of mismatches in the gRNA/target complexes, which was used to develop a matrix for predicting non-target sites 189. The presented algorithms have been incorporated into the development of such software as CRISPRScan, GuideScan, and the CRISPOR 187, 190, 191. Later, Medoza's group created their own algorithm CASPER, which can be applied to a variety of different organisms 192.

#### 5.2.2. Changing gRNA and sgRNA sequences and adding chemical modifications

**Figure 11:**
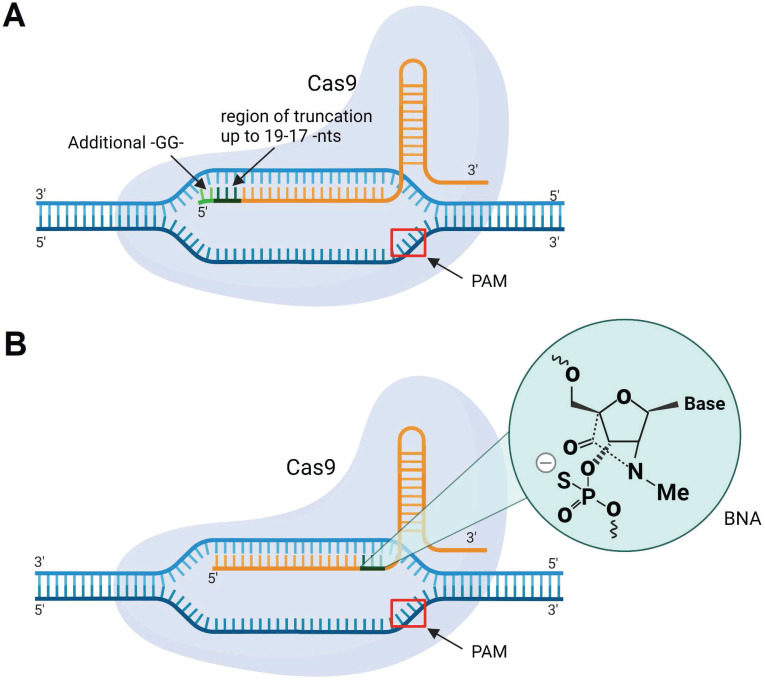
** Cas9 with sgRNA modifications. A)** sgRNA with 5'-terminal truncations (up to 19-17 nucleotides) or two guanine residues (-GG-) added to the 5'-end of sgRNA. **B)** sgRNA with incorporated BNA^NC^-modified nucleotides.

The classic design of gRNA contains a 20-nt region complementary to DNA targets. However, the gRNA/Cas9 complex can recognize and cleave the targeted DNA with a tolerance of up to 5 mismatches 193. One way to overcome mismatch-mediated HD OTE is to shorten the sequence of sgRNA to 17-18 nt from its 5' end (Figure 11A) 193. It was noted that further shortening of the spacer fragment to ≤16 nt weakens DNA binding 193. At the same time, Cho *et al*. found that an sgRNA having two additional 5'-terminal guanines (Figure 11) makes the sgRNA/Cas9 complex more specific to binding to the targeted DNA 194. Later, Cromwell *et al*. introduced bridged nucleic acids with N-methyl substitution (BNA^NC^) into gRNA (Figure 11B) 195. Three BNA^NC^ in the 3'-terminal fragment of the spacer showed improvement in DNA binding specificity (Figure 11B). However, reduction in Cas9 catalytic rates with such alterations was noticeable 195.

#### 5.2.3. Cas9 modifications

**Figure 12:**
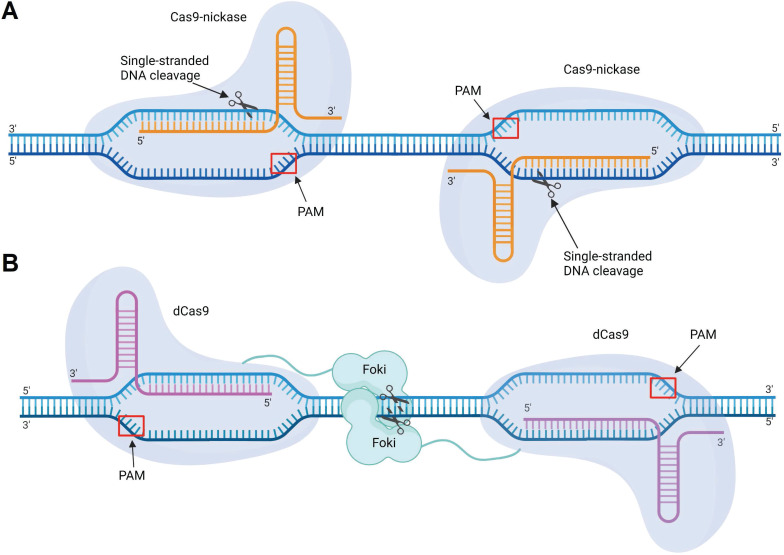
** Modifications of Cas9. A)** Cas9 nickases. Pair of Cas9 nickases generate a single-stranded break (SSB) in upstream and downstream DNA strands resulting in double stranded break. **B)** dCas9-Foki fusions. Formation of a dsDNA break requires two dCas9-Foki bound to regions close enough allow formation of a Foki dimer.

Engineered Cas9 enzymes include nickases, dCas9-nuclease fusions, and Cas9 systems with reduced OTEs. Shen *et al.* used Cas9 nickases with point mutations that inactivated either the HNH or RuvC nuclease domain (Figure 10). Each mutated nickase introduced one ssDNA break. Therefore, two nickases were required to generate a DSB (Figure 12A). This Cas9 modification showed >20-fold decrease in the sgRNA-mediated OTE in comparison with the initial Cas9 196. In another strategy, Guilinger *et al.* fused an inactive Cas9 (dCas9) enzyme with Foki nuclease domains 197. This system was similar to ZFN and TALEN with DNA-recognizing subunits fused with Foki 198. In this case, a complete DSB occured only if two Foki domains form a dimer in the cleavage site. Like Cas9 nickases, dCas9-Foki required each enzyme subunit to provide a dsDNA break (Figure 12B). This approach demonstrated >150 fold reduction in OTE 198.

Slaymaker's group modified Cas9 1.0 and 1.1 with individual alanine substitutions in RuvC lobe to neutralize its positively charged DNA-binding residues and decrease its hybridization activity 199. Later, Kleinstiver *et al.* developed Cas9 with high-fidelity (Cas9-HF1) mutated DNA-binding amino-acids in HNH and REC domains. It was reported, that on-target efficiency of Cas9-HF1 was increased by more than 85% in comparison with Cas9 200. Recently, Chen's group presented a hyper-accurate version of Cas9 (HypaCas9) with a catalytically inactive REC3 domain 201. This modification turned out to be even more accurate than the previously reported Cas9-HF1 and eCas9 201.

CRISPR/Cas has proven to be a powerful tool for genetic engineering and is still considered promising for gene therapy. However, it is heavily impacted by HD OTE. The specificity of CRISPR/Cas systems can be improved using specialized software and protein engineering tools. However, due to this limitation, the potential of CRISPR/Cas systems as *in vivo* gene therapy agents has been re-focused for an *ex vivo* application, in which gene therapy occurs on the *in vitro* followed by returning the altered cells *in vivo*. Such therapy is safer since a cell colony in the required therapeutic volume can be obtained from single successfully modified cell 202, 203. In 2022, Vertex Pharmaceutics and CRISPR Therapeutics reported CRISPR/Cas *ex vivo* based therapy (TX001) to treat patients with transfusion-dependent beta thalassemia and severe sickle cell disease 204.

## 6. Concluding remarks and future perspectives

The OGT agents discussed in this review use nucleic acid hybridization principles for target recognition, which have been thoroughly studied for hybridization probes 19, 27, 59. It was observed that conventional hybridization probes achieve high specificity only if form unstable hybrids with their targets. OGT agents, however, must tightly bind their targets to unwind mRNA secondary structures or dsDNA (in case of CRISPR/Cas). The OGT agent/target hybrids might be additionally stabilized by forming complexes with RNase H, RISC, and cas9. This tight binding quality predisposes OGT agents to have HD OTE. Interestingly, such effects were not reported for ASO agents up until 2014 or only after ~ 40 years since their introduction. In contrast, HD OTE were observed and well-studied for siRNA and CRISPR/Cas9 immediately after emerging of these technologies thus exposing the greater scale of the problem for these two technologies. This could be explained by using RNA as a target recognition probe in both cases. Indeed, RNA form more stable complexes with their targets than DNA oligonucleotides, and, therefore, such complexes tolerate mismatches a greater degree. At the same time, the 1^st^ generation of the ASO technology, which was dominating until the 21st century, used PS probes known to have low affinity to RNA, which made them more specific. Indeed, majority of HD OTE for ASO agents were reported for the LNA-containing OGT agents having high binding affinity. We make a conclusion here that LNA-based OGT are less specific, which contradicts with the popular claim of 'high LNA specificity' but agrees with both affinity/specificity dilemma and the experimental data reviewed in this work. Therefore, we agree with the earlier claims that decrease rather than increase OGT/target affinity may help reduce HD OTEs 50,51,58. Computational prediction of off- and on-target effects is a relatively developed field. However, a theoretical prediction never guarantees the desired outcome in experiment. For example, an algorithm may exclude useful sequences since the predicted HD OTEs are not always observed experimentally. In the list of OGTs, Dz agents stand out due to their ability to inhibit mRNA in a protein-independent mode. This opens an opportunity of re-designing their structures for achieving high specificity. Recent examples indicate that multicomponent ASO and Dz agents have a potential of solving the HD OTE problems 23,26,162. However, efforts are needed to convert the reported agents to a therapeutic technology.

## Dedication

The review is dedicated to the memory of the late Prof. Galina G. Karpova, one of the pioneers of antisense technology (see ref. 28 of this review).

## Figures and Tables

**Table 1:** Hybridization dependent off-target effects of antisense oligonucleotides

mRNA target	Off-target genes/effects	Comments: Cell culture/animal model/phase of clinical study	Nucleic acid modifications	Intracellular or tumor delivery/concentration	Assessment method	Ref.
Human apolipoprotein C3 (ApoC3)	Genes associated with hepatotoxicity as well as 17 genes involved in the clathrin-mediated endocytosis.	mice	LNA-DNA gapmer	25 mg/kg (5.7µM)	Microarray	41
BACH1	mRNA and protein knockdown of off-targets with a wide range of mismatch (MM) and gap patterns.	A549 and NHBE cells	LNA^a^-PS^b^ ASO^c^ gapmer^d^	unassisted or Lipofectamine 2000, Up to 50 µM	qPCR, branched DNA assay, immunoblotting	42
F7, F11 and SOD1	hepatotoxicity mediated by silencing of many unintended transcripts through RNase H1 cleavage.	mice	LNA-PS ASO gapmers, cEt-PS ASO gapmers	Subcutaneous injection	qPCR, microarray analysis, western blot	43
Nr3c, Acsl1, ApoB, Hprt1, and human Kif11	hepatotoxicity not linked with on-target activity of ASO agents, but with the RNase H1 cleavage activity.	mice	LNA-PS ASO gapmers	Subcutaneous injection	qPCR, microarray analysis, western blot	44
ApoB, Pcsk9	Significant and specific reduction of many transcripts with one- or two mismatches or bulges by RNase H1 mediated cleavage activity.	mice	LNA-PS ASO gapmers	Intravenous injection	qPCR, microarray analysis	45
Xbra, Xbra3	Splicing defects in *dtymk* and *abi1* genes contained ≥8 base pairs complementarity to PMO.	*Xenopus tropicalis*	PMO^e^		qPCR	20
Myd88	Hepatotoxic ASO-LNA demonstrated downregulation of 293 off-target genes and upregulation of 60 off-target genes and concentration dependent elevation of caspase activity in 3T3 cell line, induction of caspase activity in A549, HeLa, HepG2 cells.	3T3, A549, HeLa, HepG2 cells	LNA	Lipofectamine 2000, 3-100 nM	Caspase 3/7 activity measurements, qPCR, microarray analysis	47

*^a^*LNA - locked nucleic acid modification; *^b^*PS - phosphorothioate modification; *^c^*ASO - antisense oligonucleotide; *^d^*gapmer - ASO consisting of internal DNA gap flanked with LNA modified nucleotides; *^e^*PMO - phosphorodiamidate morpholino modified ASO.

**Table 2:** Summarized HD OTE for siRNA for cancer therapy

	mRNA target	Off-target genes/effects	Cell culture/animal model	Nucleic acid modifications	Intracellular delivery	Assessment method	Ref.
*In vitro*	IGF1R and MAPK14	IGF1R siRNAs silenced unintended targets with partial complementarity to both sense and antisense strands. MAPK14 siRNA silenced 3 genes with 13-14 nt identity of core siRNA sequence and 6 genes with 5-10 nt complementarity to 5' end of antisense strand	HeLa	-	Oligofectamine (Invitrogen)	RT-qPCR^a^ with TaqMan; microarray profiling	80
	siRNA library (particularly GRK4, BTK)	HIF-1-α with 7 nt complementarity of 3' UTR^b^ to 2-8 position in siRNA	H1299		Lipofectamine 2000	HIF-1 reporter assay, western blotting, RT-qPCR	81
	MAPK14, MPHOSPH1, PIK3CB, SOS1	Silencing of unintended transcripts with the seed region complementarity to the 3' UTR of siRNA with unique expression profile for each siRNA	HeLa	-	Oligofectamine	Microarray analysis, western blotting, RT-qPCR	82
	PLK1	33 transcripts down-regulated by the **shRNA** 20 displayed 3' UTR sequence complementarity to positions 1-6, 2-7, and 3-8 hexamers of the shRNA guide strand	Human colon cancer cells (HT29)	-	Lentiviral vector	Microarray analysis, western blotting, RT-qPCR	82
	PPIB, MAP2K1 and GAPDH	12 siRNAs silenced 347 off-targeted genes with 3' UTR matches to siRNA 2-7 or 2-8 positions of siRNA antisense strand, only 23 from them were identified *in silico*	HeLa, human embryonic kidney cells (HEK293)	-	Lipofectamine 2000	Microarray analysis	83
	Apolipoprotein B	Number of off-target transcripts enriched for 3' UTR seed hexamer matches with the largely distinct expression profiles between human and murine cells. Best-silenced transcripts are PLDN (in human cells) and BIVM (in mouse cells)	Human liver-tumor derived cells HUH7 and PLC/PRF/5, murine hepatoma cells Hepa1-6 (mouse)	2′-O-Me^c^, 2′-fluoro, 3', 5' inverted deoxy abasic, PS^d^	Lipofectamine RNAiMAX	Microarray analysis, ANOVA, RT-qPCR	85
	siRNA library (particularly FGFR2, TNFRSF13B and PRDM13)	Mcl-1 via microRNA-like mechanism	NCI-H196	-	Lipofectamine 2000	Western blotting, RT-qPCR	86
	siRNA library	TGFBR1 and TGFBR2 via miRNA-like off-target effect mechanism	HaCaT keratinocytes	-	Lipofectamine 2000	RT-qPCR, luciferase reporter assay	87
	Nucleostemin	Downregulation of 182 genes, 26 of which are transcription regulators and 56 are DNA binding proteins	Cancer stem cells from glioblastoma	-	Lentiviral vector	RT-qPCR, microarray analysis	88
	siRNA library (particularly AVPR1A and CDK5R1)	CDH1, ZEB1, KRAS, MYBL1	Pancreatic cancer cells PANC-1	-	Lipofectamine RNAiMAX	RT-qPCR	89
	CD95, CD95L	Distinct form of cancer cell death, resulted from the targeting survival genes: TFRC, NUCKS1, FSTL1, CCT3, CAPZA1, SNRPE, NAA50, FUBP1, GNB1	NB7, HeyA8, MCF-7, HCT116, 293T	-	Lentivaral vector	RNA-Seq^e^, arrayed qPCR	91
*In vivo*	Apolipoprotein B	Suppression of number of off-target transcripts	mice	2′-O-Me, 2′-fluoro, 3', 5' inverted deoxy abasic, PS	Lipid nanoparticles	Microarray, ANOVA, RT-qPCR	85

*^a^*RT-qPCR - reverse transcription quantitative real-time PCR; *^b^*3' UTR - 3' untranslated region of mRNA; *^c^*2'-O-Me - 2′-O-methyl modification; *^d^*PS - phosphorothioate modification; *^c^*RNA-Seq - RNA sequencing.

**Table 3:** Ribozymes and deoxyribozymes used for cancer suppression

Ribozymes							
	mRNA target	Type of cancer	Cell culture/animal model/phase of clinical study	Nucleic acid modifications	Tumor delivery	Off-target effects	Reference
*In vivo* (animal models)	ft-1 vascular endothelial growth factor (VEGF)	numerous human tumor types	Cynomolgus monkeys, mice	4 phosphorothioate bonds and an inverted 3'-3' deoxyabasic sugar in one of the recognition arms	intravenously, subcutaneously	None	135
	HERZYME HER-2/*neu*	breast cancer	nude mice	4 phosphorothioate bonds and an inverted 3'-3' deoxyabasic sugar in one of the recognition arms	subcutaneously	None	136
Clinical studies	ft-1 vascular endothelial growth factor (VEGF)	breast and colorectal cancer	Phase I, multidose phase I/II, phase II	4 phosphorothioate bonds and an inverted 3'-3' deoxyabasic sugar in one of the recognition arms	intravenously, subcutaneously	None	137,138
	HERZYME	breast cancer	Phase I	4 phosphorothioate bonds and an inverted 3'-3' deoxyabasic sugar in one of the recognition arms	subcutaneously	None	139
**Deoxyribozymes**							
	mRNA target	Type of cancer	Cell culture / animal model / phase of clinical study	Nucleic acid modifications	Tumor delivery	Comments	Reference
*In vivo* (animal models)	c-jun	nodular basal-cell carcinoma	primates, mice	-	intravenously	Off-target: activation of inhibitor of caspase-activated deoxyribonuclease and protein kinase C delta	144,145
	MMP-9		normal and transgenic mice	-		None	148
	EGR-1	breast carcinoma	nude mice	-	intratumoral	None	149
Clinical studies	c-jun	nodular basal-cell carcinoma	phase I	-	intratumoral	None	146
	EBV-LMP1	Nasopharyngeal carcinoma	phase I	phosphorothioate-modified “10-23” DNAzymes	intravenous administration	None	147

## Abbreviations

4WJ: 4-way junction
AAV: Adeno-associated viruses
AGO2: Argonaute 2
ASO: antisense oligonucleotides
AgoshRNA: Ago2-dependent shRNA
AmNA: amino-bridged nucleic acid
BLAST: Basic Local Alignment Search Tool
BNA^N^: bridged nucleic acids with N-methyl substitution
CDH-1: E-cadherin
CRISPR: clustered interspaced short palindromic repeats
CTGF: connective tissue growth factor
Cas: CRISPR associated protein
DNA: deoxyribonucleic acid
DSB: dsDNA break
DsiRNA: Dicer substrate interfering RNA
Dz: deoxyribozymes
FDA: United States Food and Drug Administration
GT: gene therapy
Hb: hemoglobin
HCV: hepatitis C virus
HD: hybridization dependent
HDR: homology directed repair
HF: high fidelity
HHRz: hammerhead Rz
HI: hybridization independent
HIF-1: heterodimeric transcription factor
HIV: human immunodeficiency virus 1
HypaCas9: hyper-accurate Cas9
IGF1R: insulin-like growth factor 1 receptor
KRAS: Kristen rat sarcoma proto-oncogene
LNA: locked nucleic acid
MAPK14: mitogen-activated protein kinase 14
MB: molecular beacon
MOE: methoxyethyl
MONi-RNAi: modified oligonucleotide-inducible RNAi
NHEJ: non-homologous end joining
OGT: oligonucleotide-based gene therapy
OMe: 2'-O-methyl
OTE: off-target effects
PAM: protospacer adjacent motif
PARN: Poly(A)-specific ribonuclease
PMO: phosphorodiamidate morpholinos
PNA: peptide nucleic acid
PS: phosphothioate modification
RISC: RNA-induced silencing complex
RNA: ribonucleic acid
RNA-seq: RNA sequencing
RNAi: RNA interference
RNP: ribonucleoproteins
RT-qPCR: real-time qPCR
Rz: ribozymes
SVM: support vector machine
SaCas9: Staphylococcus aureus
StCas9: Streptococcus thermophilus
TGF: transforming growth factor
TGFBR: TGF-β receptor
TLR: toll-like receptor
TRAP: targeted ribozyme-attenuated probe
Tm: melting temperature
UNA: unlocked nucleoside analog
UTR: untranslated regions
araU: 2'-F,4'-C-OMe:arabinouridine
biASO: binary ASO
cEt: constrained ethyl substituted
dsDNA: double-stranded DNA
dsRNA: double-stranded RNA
gRNA: guide RNA
mRNA: messenger RNA
miRNA: microRNA
pri-miRNA: primary RNA
pri-shRNA: primary shRNA
qPCR: quantitative PCR
sgRNA: single guide RNA
shRNA: short hairpin RNA
siRNA: small interfering RNA
sisiRNA: segmented siRNA
sshRNA: short shRNA
